# Influenza virus differentially activates mTORC1 and mTORC2 signaling to maximize late stage replication

**DOI:** 10.1371/journal.ppat.1006635

**Published:** 2017-09-27

**Authors:** Sharon K. Kuss-Duerkop, Juan Wang, Ignacio Mena, Kris White, Giorgi Metreveli, Ramanavelan Sakthivel, Miguel A. Mata, Raquel Muñoz-Moreno, Xiang Chen, Florian Krammer, Michael S. Diamond, Zhijian J. Chen, Adolfo García-Sastre, Beatriz M. A. Fontoura

**Affiliations:** 1 Department of Cell Biology, University of Texas Southwestern Medical Center, Dallas, Texas, United States of America; 2 Department of Microbiology, Icahn School of Medicine at Mount Sinai, New York, New York, United States of America; 3 Global Health and Emerging Pathogens Institute, Icahn School of Medicine at Mount Sinai, New York, New York, United States of America; 4 Department of Molecular Biology, University of Texas Southwestern Medical Center, Dallas, Texas, United States of America; 5 Department of Medicine, Washington University School of Medicine, St. Louis, Missouri, United States of America; 6 Howard Hughes Medical Institute, University of Texas Southwestern Medical Center, Dallas, Texas, United States of America; 7 Department of Medicine, Division of Infectious Diseases, Icahn School of Medicine at Mount Sinai, New York, New York, United States of America; University of Wisconsin-Madison, UNITED STATES

## Abstract

Influenza A virus usurps host signaling factors to regulate its replication. One example is mTOR, a cellular regulator of protein synthesis, growth and motility. While the role of mTORC1 in viral infection has been studied, the mechanisms that induce mTORC1 activation and the substrates regulated by mTORC1 during influenza virus infection have not been established. In addition, the role of mTORC2 during influenza virus infection remains unknown. Here we show that mTORC2 and PDPK1 differentially phosphorylate AKT upon influenza virus infection. PDPK1-mediated phoshorylation of AKT at a distinct site is required for mTORC1 activation by influenza virus. On the other hand, the viral NS1 protein promotes phosphorylation of AKT at a different site via mTORC2, which is an activity dispensable for mTORC1 stimulation but known to regulate apoptosis. Influenza virus HA protein and down-regulation of the mTORC1 inhibitor REDD1 by the virus M2 protein promote mTORC1 activity. Systematic phosphoproteomics analysis performed in cells lacking the mTORC2 component Rictor in the absence or presence of Torin, an inhibitor of both mTORC1 and mTORC2, revealed mTORC1-dependent substrates regulated during infection. Members of pathways that regulate mTORC1 or are regulated by mTORC1 were identified, including constituents of the translation machinery that once activated can promote translation. mTORC1 activation supports viral protein expression and replication. As mTORC1 activation is optimal midway through the virus life cycle, the observed effects on viral protein expression likely support the late stages of influenza virus replication when infected cells undergo significant stress.

## Author summary

Drug-resistant influenza viruses commonly arise due to frequent genetic changes and current antiviral drugs are not highly efficient. These underscore the need for new antiviral therapies effective against influenza viruses. Understanding how influenza virus uses cellular proteins for infection can potentially identify novel targets for pharmacological intervention. Influenza virus modulates cellular pathways to promote its replication and avoid immune restriction. Here we reveal the interplay between the cellular protein mTOR, which functions in two distinct protein complexes, and influenza virus infection. mTOR complex 1 (mTORC1) is activated during influenza virus infection through a cascade of specific modifications, or phosphorylation events, and by reducing the levels of another cellular protein termed REDD1, which is an mTORC1 inhibitor. Activation of mTORC1 results in additional phosphorylation events that together promote viral protein expression and replication. On the other hand, mTOR complex 2 (mTORC2) phosphorylates AKT at a specific site during infection, which is a process mediated by the viral NS1 protein that is known to regulate viral-mediated cell death. Since these effects occur midway through the virus life cycle in the infected cell, mTORC1 and mTORC2 activation are likely important to regulate the cellular environment in order to facilitate the late stages of viral infection.

## Introduction

Influenza virus is a major human pathogen for which there are few treatment options. To search for novel potential therapeutic targets while systematically investigating viral-host interactions, comprehensive proteomics screens [1,2] and various genome-wide screens were performed in influenza virus infected cells [3]. In two screens, the host kinase mechanistic target of rapamycin (mTOR) was identified as a protein that promotes influenza virus infection [4,5]. Indeed, we and others found that H1N1 influenza viruses activate mTORC1 [5,6], and influenza virus replication is reduced when mTOR is inhibited [4]. Additionally, we showed that mTORC1 inhibition by chemical induction of REDD1, a known mTORC1 inhibitor, reduced influenza virus replication [5]. Collectively, these data highlight the importance of mTORC1 for efficient influenza virus replication.

**Fig 1:**
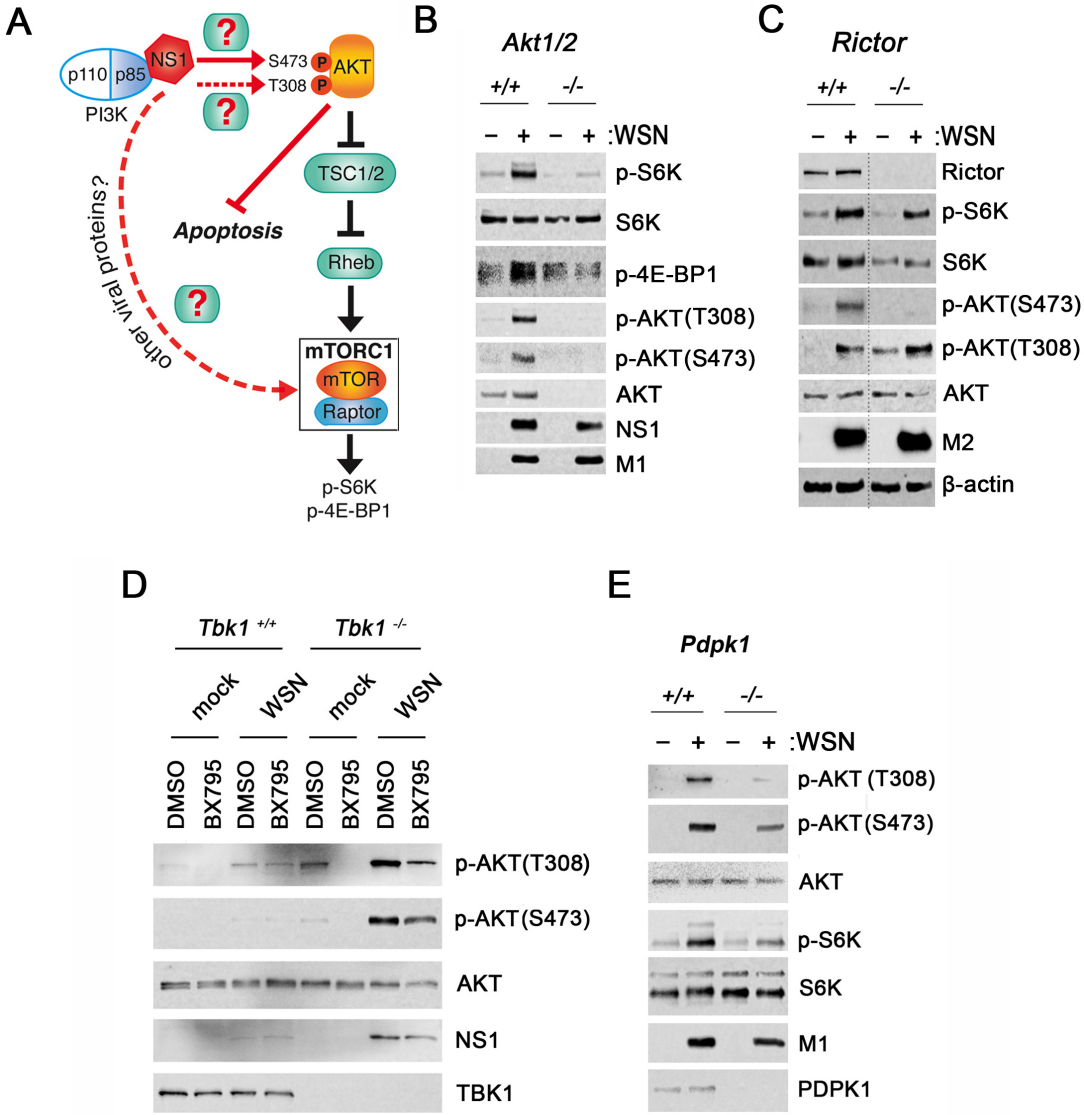
PDPK1 phosphorylates AKT to promote mTORC1 activation during influenza virus infection. (**A**) Schematic representation of the PI3K/AKT pathway and mTORC1 activation. Filled red lines represent regulation of the signaling pathway by influenza virus. Dotted lines depict gaps of knowledge in these pathways, and boxes with question marks indicate unknown regulatory factors. (**B**) HCT116 *Akt1/2*^*+/+*^ and *Akt1/2*^*-/-*^ were infected with WSN at MOI of 2 PFU/cell for 6h. Cell lysates were subjected to immunoblot analysis with antibodies against the depicted proteins. (**C**) *Rictor*^*+/+*^ and *Rictor*^*-/*-^ MEFs were processed as in **B** except that infection was performed for 10h. (**D**) *Tbk1*^*+/+*^ and *Tbk1*^*-/-*^ MEFs +/- 1 μM BX795 were processed as in **B**. (**E**) HCT116 *Pdpk1*^*+/+*^ and *Pdpk1*^*-/-*^ were processed as in **B**. Data are representative of three independent experiments. Dotted line in **C** indicates omission of unnecessary lanes from the same immunoblot membrane. M2, M1 and NS1: viral proteins; host proteins: β-actin, Rictor, TBK1, PPDPK1, total and phosphorylated S6K, AKT and 4E-BP1. Total S6K, β-actin and total AKT serve as loading controls.

mTOR is a highly conserved serine/threonine kinase that resides in two functionally distinct multi-protein complexes termed mTOR complex 1 and 2 (mTORC1 and mTORC2), which are defined by association with the proteins Raptor or Rictor, respectively. mTORC1 regulates cellular protein synthesis, growth and proliferation in response to nutrients, such as amino acids and growth factors. Amino acid stimulation promotes mTORC1 recruitment to lysosomes, which results in mTORC1 activation [7]. This process requires amino acid-sensing by the lysosomal vacuolar H^+^-adenosine triphosphatase ATPase (v-ATPase) and its interaction with the Ragulator complex of scaffold proteins [8]. The Ragulator then recruits the Rag GTPases, which brings mTORC1 in close proximity to its small GTPase activator Rheb (Ras homolog enriched in brain) [7]. Growth factors activate mTORC1 through a signaling axis involving phosphoinositide 3-kinase (PI3K) and AKT (Fig 1A). Once activated by phosphorylation, AKT inhibits Tuberous Sclerosis Complex 1/2 (TSC1/2), which is a GTPase-activating protein (GAP) that acts on Rheb, thereby stimulating mTORC1. REDD1 inhibits mTORC1 activation in a TSC1/2-dependent manner [9]. Activated mTORC1 phosphorylates downstream substrates to elicit cellular responses. p70 S6 kinase 1 (S6K) and 4E-BP1 are key mTORC1 substrates that regulate protein translation. S6K phosphorylation stimulates translation initiation and elongation through its substrate ribosomal protein S6. Phosphorylation of 4E-BP1 results in loss of eIF4E binding so that eIF4E can help facilitate cap-dependent translation initiation [7].

The proteins Rictor and SIN1 distinguish mTORC2 from Raptor-containing mTORC1. mTORC2 regulates the cytoskeleton, cell survival and has a more recently identified role in translation [10–13]. The activation and functions of mTORC2 are not as well characterized as mTORC1. Recent studies indicate that phosphatidylinositol-3,4,5-triphosphate (PIP_3_) activates mTORC2 in response to growth factors or insulin [14,15]. mTORC2 substrates include AGC kinases AKT, PKC-α and SGK1. Notably, mTORC2 phosphorylates AKT at serine 473 (S473), a growth stimuli responsive site that promotes cell survival [13]. mTORC1 and mTORC2 signaling are often enhanced during oncogenic transformation, and both are being considered as targets for cancer therapeutics [7].

The influenza virus non-structural protein 1 (NS1) has been shown to interact with the p85β subunit of PI3K, which results in activation of AKT S473 [16,17] (Fig 1A). AKT activation by influenza virus is thought to suppress apoptosis as PI3K/AKT signaling is well known to promote cell survival, and NS1 has anti-apoptotic functions [18–21]. However, NS1 proteins, especially from avian strains, were shown to have pro-apoptotic functions [22–25]. Despite the binding of NS1 to PI3K, it is not entirely clear whether this interaction contributes to apoptotic regulation [26]. It has also been hypothesized that the anti-apoptotic function of NS1 would contribute to the early stages of the virus life cycle whereas the pro-apoptotic role would be important at late stages of the infection cycle [27]. PI3K activation stimulates 3-phosphoinositide dependent protein kinase-1 (PDPK1, also known as PDK1)-mediated phosphorylation of AKT at threonine 308 (T308) [28], whereas mTORC2 and other kinases are responsible for AKT phosphorylation at S473 [29]. Notably, TANK binding kinase 1 (TBK1) phosphorylates both the T308 and S473 sites on AKT [30,31]. However, the relationship between AKT and mTOR activation during influenza virus infection has not been established. Here we sought to understand the players and mechanisms involved in mTORC1 activation during influenza virus infection and identify the mTORC1-dependent substrates and pathways activated during infection.

## Results

### PDPK1 activates AKT to promote mTORC1 activation during influenza virus infection

Influenza virus infection promotes AKT phosphorylation at amino acid S473 [16–18,32], but the kinase involved has not been identified, and phosphorylation at the T308 site has not been examined. To first determine whether AKT is required for mTORC1 activation, wild-type cells and cells lacking two of the three AKT isoforms AKT1 and AKT2 were infected. Influenza A virus strain A/WSN/1933 (WSN) induced AKT phosphorylation at S473 and T308 sites as well as mTORC1 activation in wild-type cells, as evidenced by phosphorylation of its substrate S6K on threonine 389 (p-S6K) and the multiple forms of the 4E-BP1 protein. However, mTORC1 activity was strikingly reduced in the absence of AKT1/2 (Figs 1B and S1A–S1D). Thus, we show that functional AKT is critical for proper induction of mTORC1 by influenza virus.

The influenza virus NS1 protein binds PI3K to induce AKT S473 phosphorylation by an uncharacterized kinase [26] (Fig 1A). AKT is fully activated when both T308 and S473 residues are phosphorylated [33]. PI3K activation results in T308 phosphorylation of AKT by PDPK1 [28]. Alternatively, AKT is phosphorylated at T308 and S473 by TBK1 [31] independently of PI3K and PDPK1 [30]. mTORC2 is one of several kinases that phosphorylates AKT at S473 [13]. To assess whether mTORC2 is required for influenza virus to activate AKT, we examined AKT activity in the absence of mTORC2 using mouse embryonic fibroblasts (MEF) expressing or lacking Rictor, a component required for mTORC2 complex formation and function [7]. In addition, we have knocked down Rictor in A549 cells. In both conditions, infection of Rictor expressing cells with WSN stimulated AKT phosphorylation at both T308 and S473 sites (Fig 1C, S1E–S1G Fig). In contrast, AKT phosphorylation at S473 was absent or severely reduced in WSN infected *Rictor*^*-/-*^ MEFs and in A549 cells where Rictor was knockdown, respectively (Fig 1C, S1E and S1G Fig). These results indicate that influenza virus promotes mTORC2-mediated phosphorylation of AKT at S473.

Stimulation of AKT T308 phosphorylation still occurred in *Rictor*^*-/-*^ MEFs to a similar degree as in *Rictor*^*+/+*^ cells when normalized to total AKT levels (Fig 1C and S1F Fig). We noticed higher basal levels of p-AKT (T308) in *Rictor*^*-/-*^ MEF as compared to *Rictor*^*+/+*^ cells (Fig 1C) but this was not the case in A549 cells with transient knockdown of Rictor versus control cells (S1G Fig). Nevertheless, influenza virus infection stimulated mTORC1 to a similar extent in both *Rictor*^*-/-*^ MEF and A549 cells depleted of Rictor compared to their respective controls, as evidenced by increased p-S6K levels (Fig 1C and S1G–S1J Fig). S6K phosphorylation at T389 was normalized to total S6K levels and showed that *Rictor*^*-/-*^ MEF has slightly lower levels of total S6K than *Rictor*^*+/+*^ cells but the degree of activation is similar to wild-type cells as mentioned above (Fig 1C, S1H–S1I Fig). In sum, mTORC1 was activated despite the absence or severe reduction of p-AKT(S473), indicating that full AKT activation is not required for influenza virus to trigger mTORC1. Since Rictor, as a member of the mTORC2 complex, is known to phosphorylate p-AKT (S473) in uninfected cells and we observed a lack or striking decrease of AKT phosphorylation at this site in *Rictor*^*-/-*^ infected MEFs (Fig 1C, S1E and S1G Fig), these findings indicate that influenza virus usurps mTORC2 to promote phosphorylation of AKT at S473. This phosphorylation event presumably occurs through NS1-mediated PI3K activation, which is a process that is known to regulate apoptosis [26]. Altogether, mTORC1 stimulation by influenza virus can occur independently of mTORC2-mediated activation of AKT through phosphorylation of the S473 site.

To ascertain the kinase responsible for phosphorylation on residue T308 of AKT during infection and its potential relationship with mTORC1 activation, we first infected wild-type and *Tbk1*^*-/-*^ cells with WSN in the absence or presence of BX795, an inhibitor of PDPK1 [34], TBK1 and IKKε [35]. mTORC1 activation by influenza virus still occurred in *Tbk1*^*-/-*^ cells and was down-regulated in the presence of BX795 (Fig 1D). These results indicated that TBK1 was not involved in influenza virus-mediated induction of mTORC1 activity and that another kinase inhibited by BX795 played a role in stimulating mTORC1. As expected, viral protein levels were higher in *Tbk1*^*-/-*^ cells than in *Tbk1*^*+/+*^ cells, as *Tbk1* knockout cells are more permissive to infection. mTORC1 activation was also higher in *Tbk1*^*-/-*^ cells than in *Tbk1*^*+/+*^ cells, suggesting that virus replication may activate mTORC1, a point that will be addressed below. Next, we tested cells that lack PDPK1 to assess if it had a role in mTORC1 activation by influenza virus. We observed that p-AKT(T308) phosphorylation was largely reduced in *Pdpk1*^*-/-*^ cells whereas p-AKT(S473) was slightly decreased after normalization to total AKT (Figs 1E, S1K and S1L). mTORC1 activation was inhibited in the absence of PDPK1 during infection (Fig 1E and S1M Fig). Taken together these findings and the results from *Rictor* knockout cells that activate mTORC1 to the same extent as *Rictor* wild-type cells in the presence of p-AKT(308) but in the absence of p-AKT(S473), these findings point to the AKT T308 phosphorylation site as important to stimulate mTORC1 activity during infection (Fig 1B–1E). It is possible that in *Pdpk1*^*-/-*^ cells phosphorylation of both T308 and S473 sites are required for full AKT activation, as has been shown with growth factor stimuli [33]; therefore, these AKT phosphorylation sites may influence the phosphorylation status of each other in certain conditions.

Altogether, mTORC2 and PDPK1 are the kinases responsible for AKT S473 and T308 phosphorylation, respectively, during infection. AKT is required for influenza virus to activate mTORC1 and AKT phosphorylation at T308 appears to be the key to trigger mTORC1 during infection, indicating that site-specific phosphorylation of AKT directs its downstream functions.

### mTORC1 activation by influenza virus is independent of the viral NS1 protein

**Fig 2:**
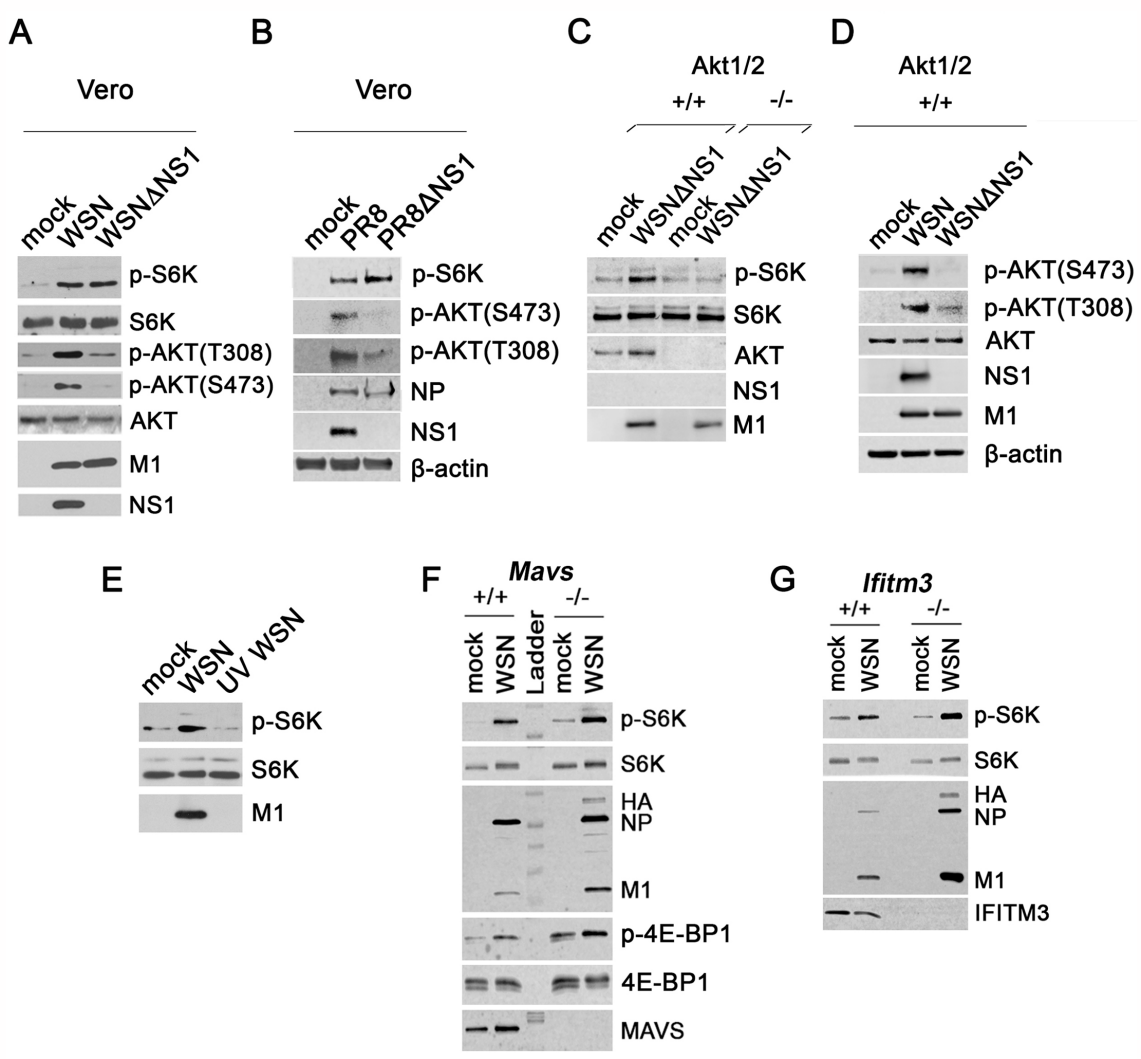
Viral replication is required for influenza virus to activate mTORC1 independently of NS1. (**A**, **B**) Vero cells were infected with (**A**) WSN or (**B**) PR8 wild-type or mutant viruses lacking NS1 at MOI of 2 PFU/cell for 6 h. (**C**,**D**) HCT116 *Akt1/2*^*+/+*^ and *Akt1/2*^*-/-*^ were infected with WSNΔNS1 or WSN at MOI of 5 PFU/cell for 10h. (**E**) A549 cells were infected with WSN or UV-inactivated WSN at MOI of 2 PFU/cell for 6h. (**F**) *Mavs*^*+/+*^ and *Mavs*^*-/-*^ or (**G**) *Ifitm3*^*+/+*^ and *Ifitm3*^*-/-*^ MEFs were infected with WSN at an MOI of 2 PFU/cell for 6 h. Immunoblot analyses were performed for detection of viral proteins (NS1, HA, NP and M1) and host proteins (β-actin, MAVS, IFITM3 as well as total and phosphorylated S6K, AKT and 4E-BP1). β-actin and total S6K serve as loading controls. The upper band in the S6K/p-S6K blots is p85 S6K whereas the lower band is p70 S6K. Data are representative of three independent experiments.

We demonstrated that influenza virus stimulates mTORC1 signaling through activation of AKT via the T308 site (Fig 1B–1E). Therefore, we sought to elucidate how AKT activation by influenza virus results in mTORC1 activity. We first examined the role of the viral NS1 protein, which is a multifunctional virulence factor that is synthesized during replication and usurps several host signaling pathways. NS1 promotes AKT S473 phosphorylation through its interaction with PI3K [26]. To determine whether NS1 is necessary for mTORC1 activation, we tested viruses lacking NS1 (WSNΔNS1 and PR8ΔNS1). NS1-deficient viruses have replication defects in cells with functional type I interferon (IFN) signaling; therefore, Vero cells were used which lack type I IFN genes [36–38], and thus allow comparable replication of wild-type and ΔNS1 viruses. mTORC1 was similarly activated by both wild-type and ΔNS1 viruses, demonstrating that influenza virus does not require NS1 to activate mTORC1 (Fig 2A and 2B). The results suggested that low levels of AKT T308 phosphorylation induced by WSNΔNS1 was sufficient to activate mTORC1 (Fig 2A, 2B and S2A Fig), whereas AKT S473 phosphorylation was severely reduced and likely not required (Fig 2A, 2B and S2B Fig). These findings are corroborated by infection of *Akt1/2*^*+/+*^ and *Akt1/2*^*-/-*^ cells with WSN or WSNΔNS1, which show that AKT is required for WSNΔNS1 induction of mTORC1 activity (Fig 2C) and that, as in Vero cells, WSNΔNS1 infected *Akt1/2*^*+/+*^ cells promoted mTORC1 activity with low levels of p-AKT(T308) and in the absence or barely detectable levels of p-AKT(S473) (Fig 2D). Therefore, mTORC1 activation by influenza virus appears to be independent of AKT S473 phosphorylation and NS1.

### Diverse influenza virus strains activate mTORC1

To clarify our understanding of viral factors involved in mTORC1 activation, we first set out to better characterize mTORC1 signal induction during influenza virus infection. To understand the kinetics of mTORC1 activation during infection, S6K phosphorylation was examined at multiple times post-infection. mTORC1 activation was evident at 4 hours post-infection and increased over time (S3A Fig). To ensure that influenza virus activates mTORC1 in non-transformed cells, mTORC1 activation was assessed in primary MEFs and human bronchial epithelial cells (HBECs). Indeed, influenza virus activated mTORC1 in primary MEFs (S3B Fig) and HBECs (S3C Fig).

We and others have previously shown that mTORC1 is activated by WSN [5,6] and A/TX/36/1991 H1N1 influenza viruses [5]. To determine whether a more distantly-related strain of influenza virus could stimulate mTORC1, cells were infected with the recently emerged H7N9 strain A/Shanghai/1/2013 (Sh/1). Sh/1 activated mTORC1 (S3D Fig). We also tested a recombinant strain that contains HA and NA segments from A/PR/8/1934 (PR8) and the other 6 segments from A/Shanghai/1/2013 (rSh/1) [39]. rSh/1 activated mTORC1 (S3E Fig). Moreover, to understand whether mTORC1 activation was a general effect of viral gene expression, we examined whether another negative-sense RNA virus vesicular stomatitis virus (VSV) was capable of activating mTORC1. VSV did not activate mTORC1 at 6 hours post-infection despite high levels of M protein expression (S3F Fig). It was previously observed that mTORC1 was activated during VSV infection in MEFs at 16 hours post-infection [40], so it appears that the kinetics of mTORC1 activation by VSV differ and might be cell type-dependent. Altogether, these results show that different strains of influenza A viruses stimulate mTORC1 signaling and that mTORC1 can be activated by influenza virus in several cell lines, highlighting the importance of mTORC1 during influenza virus infection.

### Viral replication is required for mTORC1 activation by influenza virus

To further elucidate the mechanism behind influenza virus-mediated mTORC1 activation, we set out to identify viral and cellular factors that might contribute to mTORC1 signaling. We first tested the M2 protein for a role in mTORC1 activation as it is implicated in regulating macroautophagy (referred to herein as autophagy) [41]. Influenza virus M2 protein induces autophagy early in infection but then inhibits autophagosome-lysosome fusion, which is necessary to degrade contents [41]. Autophagy inhibition can lead to mTORC1 activation as they are antagonistic processes [7]. To decipher whether M2 restriction of autolysosomal degradation can induce mTORC1 activity, we infected cells with a viral mutant strain that is deficient in M2 expression (PR8:WSNDeficientM2) and the wild-type strain (PR8:WSN). The mutant virus is propagated in an M2 expressing cell line and therefore contains M2 in virions, which is necessary for entry, but is unable to synthesize new M2 protein [41]. Both wild-type and mutant virus strains activated mTORC1 similarly, despite the lack of autophagy in PR8:WSNDeficientM2 infected cells as determined by LC3-I to LC3-II conversion (S4A Fig). Therefore, mTORC1 stimulation by influenza virus is independent of M2 effects on autophagic processing. We further evaluated a possible role of autophagy in mTORC1 induction as mTORC1 can be reactivated in cells that are serum starved for 6 or more hours, which requires initial autophagy induction that is eventually reduced due to autolysosome degradation [42]. Autophagy is induced early during influenza virus infection [43], but discontinuation of autophagy could promote mTORC1 activation. To establish whether autophagy is required for mTORC1 activation during influenza virus infection, we infected cells depleted of or lacking major proteins involved in autophagosome formation, Atg5 and Atg7. Depletion of Atg5 or Atg7 did not alter mTORC1 activation by influenza virus (S4B Fig). Furthermore, mTORC1 was still activated by influenza virus in *Atg5*^*-/-*^ cells (S4C Fig). Thus, induction and termination of autophagy does not contribute to mTORC1 activation during influenza virus infection. Overall, we demonstrated that NS1 and autophagy are dispensable for influenza virus to stimulate mTORC1 signaling.

To investigate other factors and processes necessary for mTORC1 activation by influenza virus, we determined what stages of the virus replication cycle were critical for mTORC1 stimulation. We first assessed whether influenza virus replication was a requirement. WSN was UV-inactivated, which was confirmed by plaque assay (S4D Fig). UV-inactivated influenza virus was unable to activate mTORC1 (Fig 2E), illustrating that influenza virus replication is likely required to stimulate mTORC1. Thus, we conclude that stimulation of mTORC1 by influenza virus is not mediated by virus-induced processes prior to viral fusion, such as binding to cellular receptors and endocytosis, and likely requires active viral replication. To further evaluate the effect of viral replication on mTORC1 activation, we tested whether mTORC1 activity was increased in cells deficient in immune signaling or effector molecules that allow enhanced virus replication compared to wild-type cells. Additionally, we sought to determine whether key innate immune proteins involved in type I IFN induction contribute to mTORC1 activation since IFN can stimulate mTORC1 [44]. To this end, MEFs deficient in MAVS and IFITM3 were utilized. MAVS is the key adaptor molecule of the RIG-I-like receptor signaling pathway, which responds to viral RNA detection and induces IFN expression in virally-infected cells [45]. Deletion of MAVS reduces innate immune responses to viral infection, which results in increased virus replication. IFITM3 is an IFN-stimulated gene that restricts influenza virus replication [46] by preventing virus escape from endosomes during entry [47]. Influenza virus protein levels and mTORC1 activation were increased in both *Mavs*^*-/-*^ and *Ifitm3*^*-/-*^ MEFs compared to their wild-type counterparts (Fig 2F and 2G), suggesting that virus replication amplifies mTORC1 signaling. Moreover, these data provide additional evidence that mTORC1 activation by influenza virus is not a result of the type I IFN response to viral infection, in agreement with our results showing mTORC1 activation by influenza virus in Vero cells, which lack type I IFN genes (Fig 2A and 2B). In addition, no mTORC1 activation was detected upon stimulation of cells with poly(I:C), which led to interferon expression (S4E–S4G Fig).

**Fig 3:**
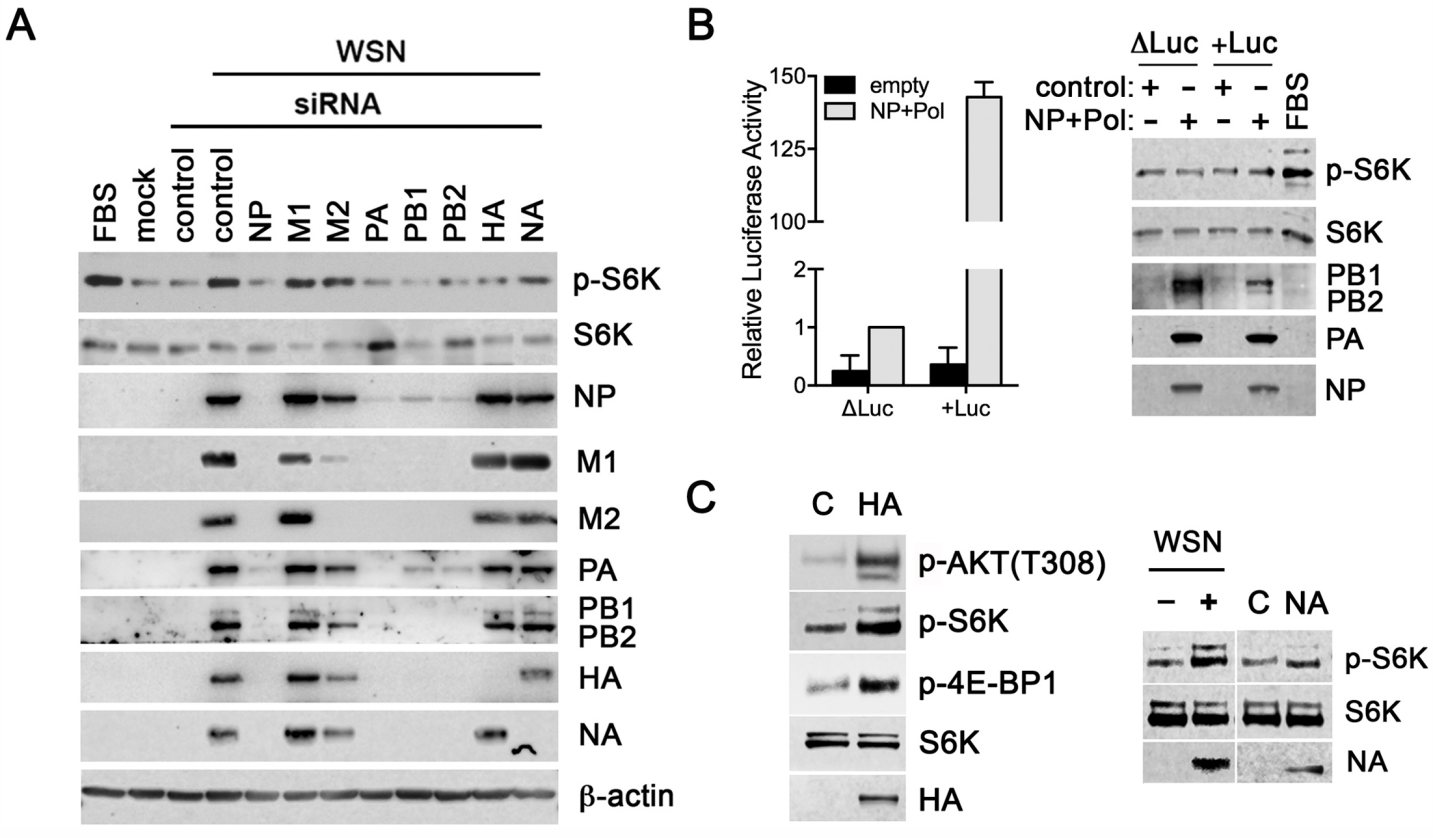
The viral protein HA promotes mTORC1 activation. (**A**) A549 cells were transfected with siRNAs (pool of three each) targeting viral mRNAs and then infected for 6 h at MOI of 2 PFU/cell. Immunoblot analysis was performed against the depicted proteins. (**B**) The activity of reconstituted polymerase complexes was measured by transfecting cells with a control plasmid or plasmids encoding NP, the polymerase subunits (PA, PB1 and PB2) and luciferase reporter genes to measure influenza virus promoter activity (mini-genome assay). Luciferase assay and immunoblot analysis were performed with antibodies against the depicted proteins. (**C**) MDCK cells were transfected with control plasmids or with plasmids encoding HA or NA. MDCK cells were also mock infected or infected with WSN at MOI of 2 PFU/cell for 6h. Immunoblot analysis were performed with antibodies against the depicted proteins. Data are representative of three independent experiments.

To investigate the potential role of additional viral proteins on mTORC1 activation, an RNA interference (RNAi) approach was taken. Small-interfering RNAs (siRNAs) against individual viral mRNAs were employed to reduce specific viral protein levels followed by assessment of mTORC1 activation. Viral siRNAs were specific to their targets with the exception of M2, which reduced both M1 and M2 (Fig 3A) since they are generated from the same RNA. Depletion of either M1 or M2 did not impact mTORC1 activity at 6h post-infection, although high levels of M2, which occur late in infection, can activate mTORC1 as addressed below. As expected, significant reductions in viral protein expression were observed when the viral polymerase complex was depleted using siRNAs against NP, PA, PB1 or PB2 (Fig 3A). Depleting the viral polymerase complex prevented mTORC1 activation likely by reducing viral replication. Indeed, when an influenza virus replication simulation system was applied to test mTORC1 activation in the presence of only NP, the polymerase complex and a mini-genome encoding negative-sense luciferase inserted in the NP cRNA promoter [48], mTORC1 was not activated (Fig 3B), suggesting that additional viral components are necessary to stimulate mTORC1. However, we did observe that knock down of HA or NA during infection reduced mTORC1 activation, although expression of other viral proteins was detectable at 6 hours post-infection (Fig 3A). Knock down of HA decreased mTORC1 activity more effectively than NA knock down. These data were corroborated by expression of HA alone, which induced AKT T308 phosphorylation and mTORC1 activation, while expression of NA alone did not promote mTORC1 activation to a significant degree (Fig 3C). Taken together, the viral protein HA promotes mTORC1 activation during infection.

### Influenza virus down-regulates REDD1 when mTORC1 activity is enhanced

**Fig 4:**
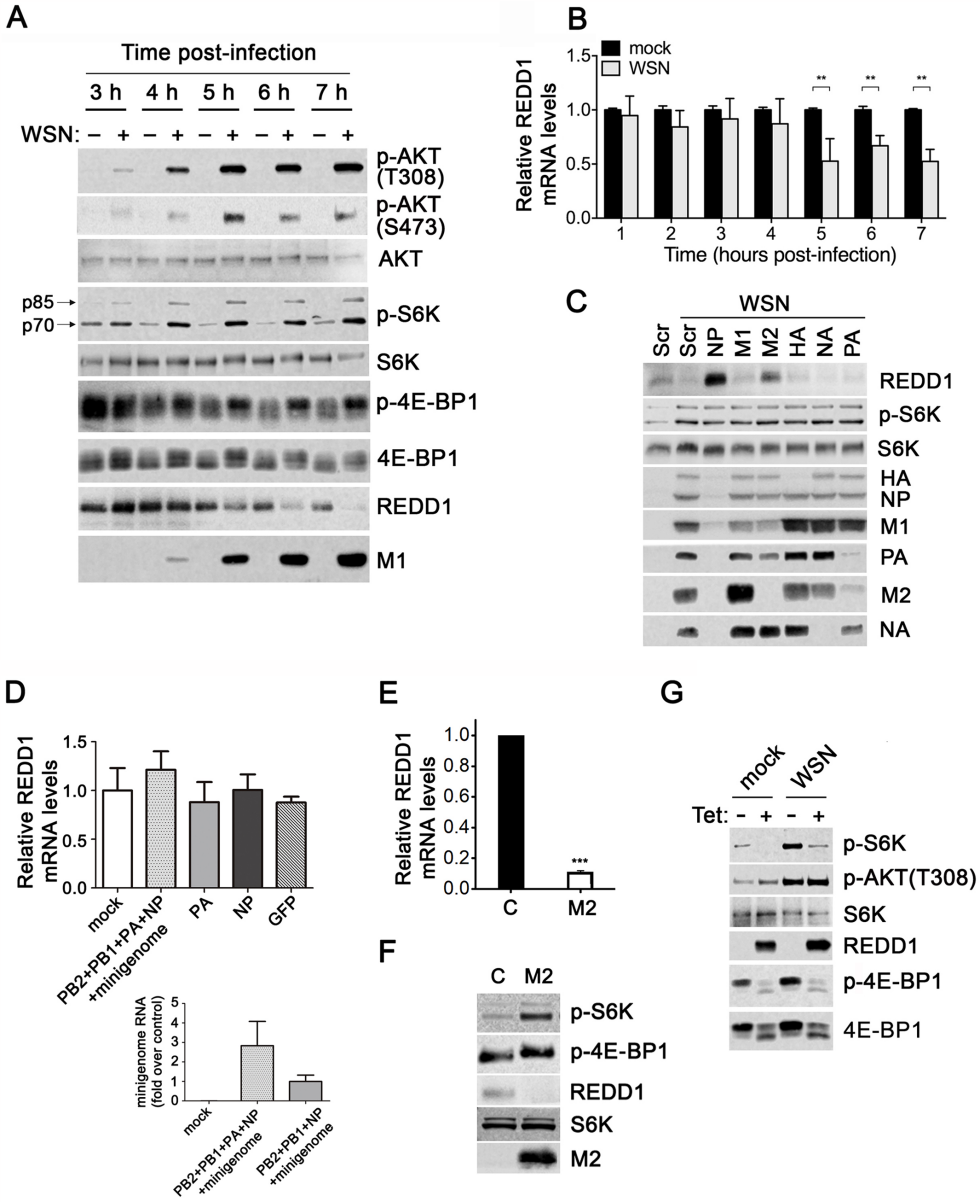
Influenza virus down-regulates REDD1 to promote mTORC1 activity. (**A-C**) A549 cells were infected with WSN at MOI of 2 PFU/cell for the indicated times. Cell extracts were subjected to (**A**) western blot analysis to detect the depicted proteins and quantified as shown in S5 Fig or (**B**) RNA was purified for qRT-PCR to determine REDD1 mRNA levels. Mean and standard deviation are shown for qRT-PCR, *n* = 4 independent experiments done in triplicates. **p<0.000004, Student's *t*-test. (**C**) A549 cells were transfected with siRNAs (pool of three each) targeting viral mRNAs and then infected for 7 h at MOI of 2 PFU/cell. Immunoblot analysis was performed to detect the depicted proteins, *n* = 3. (**D**) A549 cells were transfected with plasmids encoding the indicated virus proteins. At 48 h post-transfection, total RNA was purified and REDD1 mRNA levels were determined by qRT-PCR as in **B**. The bottom panel in **D** shows viral polymerase activity upon tranfection of the depicted viral proteins and/or minigenome as control. Minigenome mRNA was measured by qRT-PCR. In cells transfected with the complete set of plasmids that encode the viral polymerase we detect the minigenome RNA transcribed by pol I directly from the plasmid in addition to the minigenme RNA amplified by the influenza proteins, indicating protein activity. In cells transfected with the same plasmids except for PA, we only detect the minigenome RNA transcribed by pol I directly from the plasmid, and the average values was set to 1. The minigenome RNA level is higher when all plasmids were transfected (*n* = 3). (**E, F**) MDCK cells were transfected with control plasmid of plasmid enconding the M2 protein. In **E,** RNA was purified for qRT-PCR to determine REDD1 mRNA levels as in **B**, *n* = 3, ***p<0.001. In **F,** cell extracts were subjected to western blot analysis to detect the depicted proteins (*n* = 3). (**G**) U2OS-REDD1 cells were treated with vehicle or 1μg/ml tetracycline for 2 h prior to and during infection to induce REDD1 expression. Cells were infected at MOI of 2 PFU/cell for 6 h. Immunoblot analyses were performed to detect the depicted proteins. Total S6K serves as the loading control. The upper band in the S6K/p-S6K blots is p85 S6K, whereas the lower band is p70 S6K (*n* = 3).

We then investigated how the AKT-TSC1/2-Rheb axis might be regulated during infection to prompt mTORC1 signaling by assessing REDD1, which converges on and inhibits the pathway. Under stress conditions, TSC2 activity is induced by REDD1, and therefore, REDD1 is inhibitory to mTORC1 [7]. REDD1 was originally described to promote TSC2 stability and activity by sequestering the TSC1/2 inhibitory protein 14-3-3 [49]. However, a recent study revealed that REDD1 promotes AKT T308 dephosphorylation to prevent mTORC1 signaling [50]. We previously identified REDD1 as an antiviral factor and showed that REDD1 protein levels decrease as viral infection progresses [5]. Thus, we analyzed how REDD1 influences AKT-mTORC1 signaling during influenza virus infection. We assessed AKT and mTORC1 activation simultaneously with REDD1 protein levels during viral infection and found that mTORC1 activity was strongest (as compared to the mock counterpart for each time point normalized to total S6K levels) when REDD1 levels were greatly diminished and AKT T308 phosphorylation was strongest by 7h post-infection (Fig 4A and S5 Fig). AKT S473 activation increased up to 5h post-infection but decreased at later time points (Fig 4A and S5 Fig). Influenza virus infection reduced REDD1 mRNA levels at and beyond 5 hours post-infection (Fig 4B), which likely contributed to the decrease in protein levels (Fig 4A and S5 Fig). REDD1 protein levels were also reduced in mock infected cells at later times post-infection, though not as great as in infected cells, which was likely a result of REDD1 having a short half-life (~5 minutes) [51,52] and the experiments being performed in serum-free media (Fig 4A and S5 Fig). mTORC1 activation was also assessed by p-4E-BP1, which shows the expected phosphorylation pattern of activation peaking at 6h post-infection (Fig 4A and S5 Fig). AKT T308 phosphorylation preceded REDD1 down-regulation by influenza virus suggesting that AKT activation is the initial stimulus for mTORC1 and that influenza virus-induced reduction of REDD1 amplifies and/or maintains mTORC1 signaling throughout infection.

To investigate what triggers down-regulation of REDD1 at the mRNA and protein levels during infection, we knocked down influenza virus proteins and assessed REDD1 protein levels. As expected, REDD1 protein levels decreased upon infection but dramatically increased upon knockdown of the viral NP protein, which prevents virus replication and viral protein expression, as NP functions with the virus polymerase (Fig 4C). These results suggest that REDD1 levels are up-regulated upon viral entry and/or during primary transcription and are down-regulated after these early processes. This is consistent with our previous results [5] and with Fig 4A in which REDD1 levels increase early in infection and are down-regulated at later stages of infection. Depletion of HA, NA, M1 and PA did not alter REDD1 protein levels. However, depletion of M2 increased REDD1 protein levels (Fig 4C). Since both NP and M2 knockdown up-regulated REDD1 protein levels, we analyzed REDD1 mRNA levels upon expression of NP alone or in combination with the viral polymerase as well as upon M2 expression alone. We found that neither NP nor components of the viral polymerase complex altered REDD1 mRNA levels (Fig 4D). However, M2 expression alone decreased REDD1 mRNA levels and induced mTORC1 activation as noted by increased levels of p-S6K and change in mobility of p-4E-BP1 (Fig 4E and 4F). Together, these findings suggest that the REDD1 down-regulation during infection is likely mediated by the viral M2 protein and that the observed NP effect on REDD1 protein levels is due to inhibition of virus replication that prevented expression of M2 protein. We have not noticed an impact of M2 on mTORC1 activation before or at 6h post-infection (Fig 3A), which is mediated by the HA protein. This is consistent with an effect of M2 at late stages of infection when REDD1 down-regulation is prominent, after 7h, and high levels of M2 protein would be present to activate mTORC1 and to further support virus replication.

To determine whether increased levels of REDD1 can reduce AKT T308 phosphorylation during infection as was previously observed under certain basal conditions [50], we employed a tetracycline-inducible REDD1 cell line, U2OS-REDD1. High levels of REDD1 did not reduce AKT T308 phosphorylation in infected cells (Fig 4G). Therefore, REDD1 does not act on AKT to implement its mTORC1 inhibitory effects during influenza virus infection. We also determined whether high levels of REDD1 could prevent influenza virus from activating mTORC1. Indeed, REDD1 over-expression inhibited mTORC1 signaling in infected cells (Fig 4G), indicating that mTORC1 is stimulated when REDD1 is down-regulated by influenza virus. Overall, we reveal that influenza virus reverses REDD1 inhibition of mTORC1 downstream of AKT to enhance mTORC1 signaling.

### mTORC1 is required for optimal influenza virus protein expression

**Fig 5:**
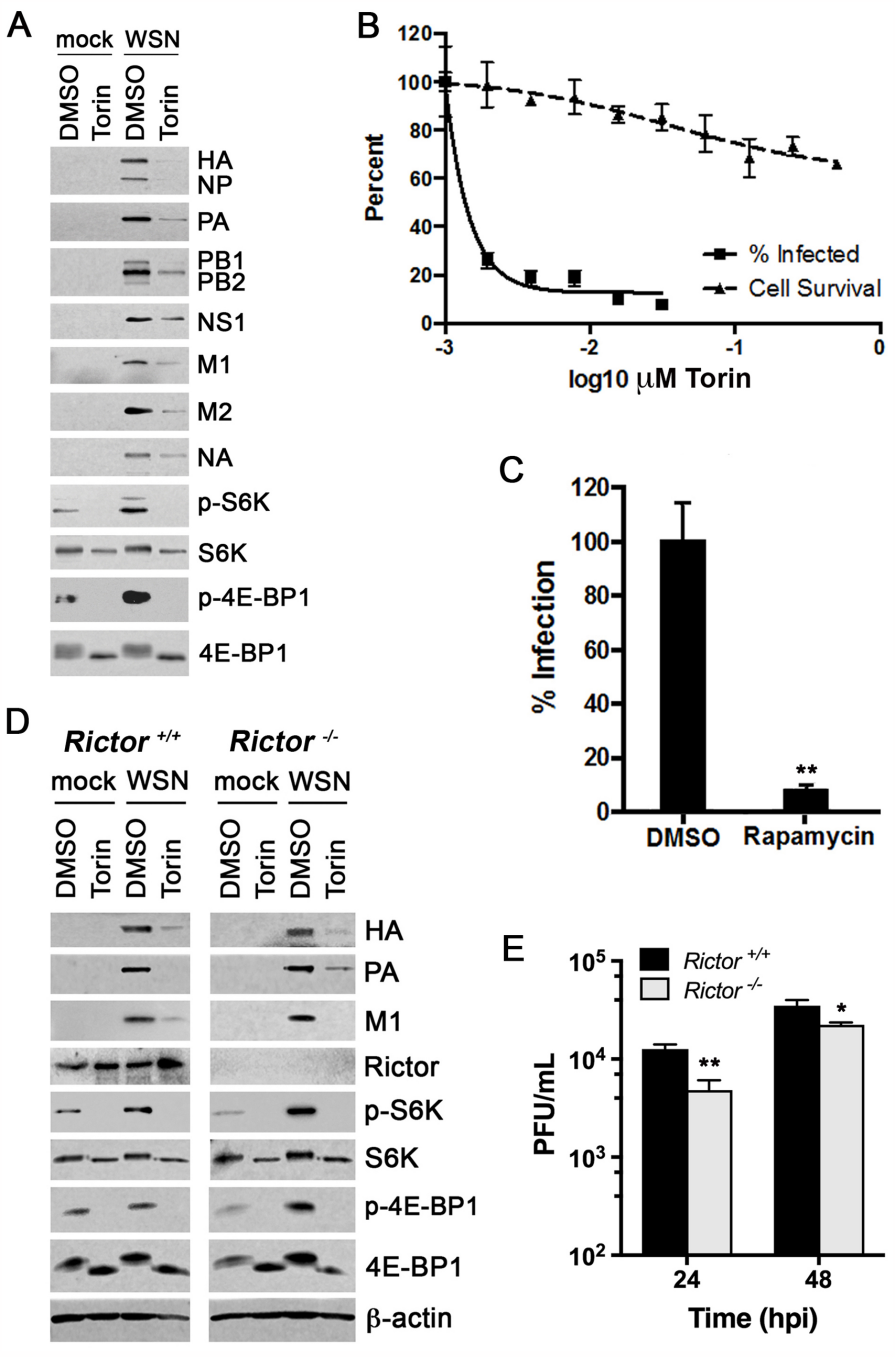
mTORC1 activation promotes viral infection. (**A**) A549 cells were infected with WSN at MOI of 2 PFU/cell for 1 h and then treated with 250 nM Torin1 or DMSO for an additional 9 h followed by immunoblot analysis. (**B**) A549 cells were infected at MOI of 0.01 PFU/cell with WSN for 16 h in the presence of the depicted concentrations of Torin1 or DMSO. Viral titers were measured by plaque assay to yield plaque-forming units per mL (PFU/ml, plotted as % infection relative to DMSO control). Cell survival was also assessed at the depicted concentrations using the MTT assay. The following parameters were calculated: CC_50_>500 nM; IC_50_ = 0.46nM; and SI>1087. (**C**) Experiment was performed as in **B** except that cells were treated with 13.7 μM rapamycin or DMSO as control. Mean and standard error of the mean (SEM) are shown. *n* = 3, **p<0.01. (**D**) *Rictor*^*+/+*^ or *Rictor*^*-/-*^ MEFs were infected with WSN at MOI of 2 PFU/cell for 6 h and treated with 250 nM Torin1 or DMSO at 1 h post-infection. Immunoblot analyses were performed to detect viral proteins (HA, NP, PA, PB1, PB2, NS1, M1, M2 and NA) and host proteins (β-actin, Rictor, total and phosphorylated S6K and 4E-BP1). β-actin and S6K serve as loading controls. The upper band in the S6K/p-S6K blots is p85 S6K whereas the lower band is p70 S6K. (**E**) *Rictor*^*+/+*^ or *Rictor*^*-/-*^ MEFs were infected with WSN at MOI of 0.01 for 24 h and 48 h. Viral titers were measured by plaque assay to yield plaque-forming units per ml (PFU/ml). Mean and standard deviation (SD) are shown, *n* = 3, **p<0.01, *p<0.05.

mTORC1 and mTORC2 signaling are enhanced during influenza virus infection (Fig 1), and both can influence translation [53]. mTORC1 promotes translation of 7-methylguanosine capped mRNAs [7], which would likely affect influenza virus protein translation as its mRNAs possess 7-methylguanosine caps acquired from host mRNAs. mTORC2 co-translationally stabilizes proteins via phosphorylation by associating with ribosomes, but few substrates have been identified thus far [53]. Therefore, we evaluated whether mTORC1 and mTORC2 can both impact influenza virus protein expression and replication. To investigate the role of mTOR on influenza virus protein expression, infected A549 cells were treated with non-toxic concentrations of the potent mTOR inhibitor Torin1 (S6A Fig), which targets both mTORC1 and mTORC2 [54]. Viral protein levels were reduced and viral mRNA levels decreased to a lesser extent in the presence of Torin1 at 10 hours post-infection (Fig 5A and S6B Fig), indicating that mTOR activity is important for optimal influenza virus mRNA and protein production. Therefore, we assessed the effect of Torin1 on the replication of WSN (Fig 5B) and rSh/1 (S6C Fig). As predicted, replication of both strains was reduced when mTOR was inhibited with Torin1. In addition, inhibition of mTORC1 by rapamycin also impaired virus replication (Fig 5C).

Torin1 does not distinguish between mTOR associated with mTORC1 or mTORC2 [54], and the inhibitor rapamycin inhibits mTORC1 but does not completely abrogate mTORC1 activity [54,55], and can inhibit mTORC2 with prolonged treatment [56]. Since mTORC1 and mTORC2 can both regulate translation [53] and are both inhibited by Torin1 [54], we tested whether Torin1-mediated reduction of viral proteins involved inhibition of both complexes by using MEFs lacking Rictor to disrupt mTORC2. If Torin1 inhibition of viral protein expression was dependent on mTORC2, then Torin1 treatment in *Rictor*^*-/-*^ MEFs would not reduce viral protein production as compared to *Rictor*^*+/+*^ MEFs. However, we observed reduced viral protein levels in both *Rictor*^*+/+*^ and *Rictor*^*-/-*^ MEFs treated with Torin1 (Fig 5D). Thus, influenza virus mainly relies on mTORC1, not mTORC2, for viral protein expression. However, mTORC2 may influence viral protein expression at different times post-infection than what was assessed here. Therefore, we assessed viral replication in *Rictor*^*-/-*^ cells compared to *Rictor*^*+/+*^ cells and we found no striking difference in virus replication (Fig 5E). Similarly, knock down of Rictor in A549 cells did not substantially affect virus replication as compared to control cells (S6D Fig). These findings together with the results above indicate PDPK1-mediated AKT phosphorylation at T308 leads to mTORC1 activation to regulate viral protein expression and replication.

### Phosphoproteomic analysis reveals mTORC1–dependent regulation of influenza virus infection

To identify what host processes are affected by influenza virus-induced mTORC1 signaling, we identified the landscape of mTORC1-dependent substrates during infection using an unbiased systematic phosphoproteomics approach in cells lacking functional mTORC2 treated with or without the mTOR inhibitor Torin1 to distinguish mTORC1 from mTORC2 substrates. *Rictor*^*-/-*^ MEFs were infected with WSN in the absence or presence of Torin1 treatment, lysed 7 h post-infection and subjected to phosphopeptide enrichment followed by mass spectrometric analyses.

**Fig 6:**
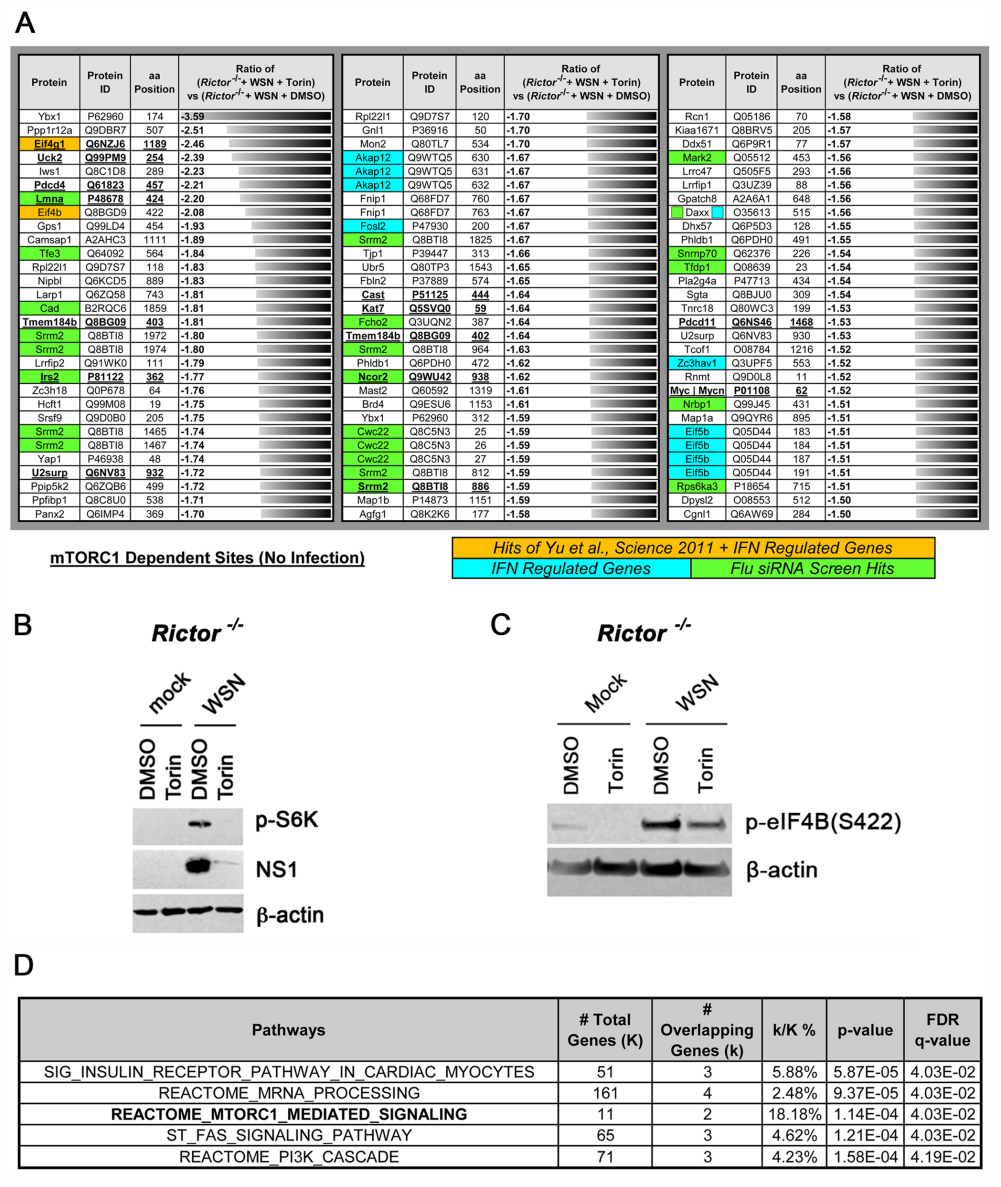
mTORC1-dependent phosphorylation identified during influenza virus infection. (**A**) *Rictor*^*-/-*^ MEFs were infected with WSN for 7 h at MOI of 2 PFU/cell and treated with Torin1 or DMSO. Cells were subjected to phosphopeptide enrichment and mass spectrometry. Data shown are results from WSN-infected *Rictor*^*-/-*^ MEFs treated with Torin1 compared to WSN-infected *Rictor*^*-/-*^ MEFs treated with DMSO. The table indicates protein names and IDs, position(s) of amino acid (aa) phosphorylation, and the ratio of phosphopeptide reads (> and < 1.5 fold or more) in Torin1-treated vs. DMSO-treated infected cells. Underlining + bold hits depicts mTORC1-dependent phosphorylation on proteins identified in mock-infected *Rictor*^*-/-*^ MEFs -/+ Torin1 (S1 Table). The protein hits that overlapped with known protein hits from other studies are depicted at the bottom of the table. (**B**) Cell lysates from *Rictor*^*-/-*^ MEFs were subjected to immunoblot analyses to confirm that Torin1 inhibited mTORC1, as shown by reduction of p-S6K, and influenza virus protein expression assessed by NS1 protein levels. (**C**) Cell lysates from *Rictor*^*-/-*^ MEFs were subjected to immunoblot analyses to show one of the hits in **A**, p-eIF4B (S422), which is down-regulated by Torin 1 and is a mTORC1 substrate in uninfected and infected cells. (**D**) Gene set enrichment analysis (GSEA) was performed for all the proteins listed in Fig 6A. The top five pathways are shown with the percentage overlap between total genes (K) in the pathway versus overlapping genes (k) identified in the our phosphoproteomics screen. *p*-values for statistical significance and *q*-values for false discovery rates (FDR) are shown.

We set out to identify mTORC1 substrates by comparing WSN infected *Rictor*^*-/-*^ MEFs treated with DMSO or Torin1. *Rictor*^*-/-*^ MEFs lack functional mTORC2, and therefore, Torin1 treatment will only inhibit mTORC1 signaling in these cells. We identified 90 phosphorylation sites that were altered +/- 1.5-fold or greater in the presence and absence of mTORC1 signaling (Fig 6A). Torin1 efficiently inhibited mTORC1 in *Rictor*^*-/-*^ MEFs (Fig 6B). Although we did not identify p-S6K(T389) or p-4E-BP1(T37/46) sites due to low peptide abundance, the S6K substrate eIF4B(S422) [57] and another previously identified mTORC1 substrate, eIF4G1(S1189), were found (Fig 6A and 6C). Both eIF4G1 (S1189) and eIF4B(S422) have been identified in an mTORC1 substrate screen [58] and we now show that these translation factors are mTORC1 substrates regulated during influenza virus infection. When comparing uninfected DMSO- and Torin-treated *Rictor*^*-/-*^ cells, nearly 16% of phosphorylation sites that we identified are previously defined mTORC1-dependent sites (Fig 6A and S1 Table). In addition to eIF4G1(S1189) and eIF4B(S422) phosphorylation changes in infected cells, LARP1(S743) phosphorylation was also altered. LARP1 was previously shown to interact with mTORC1 [59,60] and stabilizes its mRNA [61].

Next, to examine whether any of the mTORC1-dependent substrates were previously identified as important host proteins in multiple genome-wide genetic screens from influenza virus infected cells, we assessed overlap between our phosphoproteomics hits and previous screen hits [4,46,62–66]. We found that 13 out of 72 (18%) of the proteins we identified as mTORC1 substrates during infection overlapped with proteins that alter influenza virus infection [3]. Since many proteins from the multiple influenza virus siRNA screens were IFN regulated, we also examined whether any of the mTORC1 substrates that we identified were IFN regulated proteins. Our phosphoproteomics analysis show that 10 mTORC1-dependent phosphorylation sites are present in proteins that are regulated by IFN. Since Torin inhibits virus replication, some of the regulation shown here could be due to diminished replication. However, the mTORC1-mediated changes in phosphorylation during infection in mTORC1 substrates identified in the absence of infection likely reflect the true role of mTORC1 as regulator of infection.

To determine the cellular processes altered by these mTORC1-dependent phosphorylation events, we assessed the hits by Gene Set Enrichment Analysis to find commonly altered pathways. We identified pathways that regulate mTORC1 signaling and/or are regulated by mTORC1 such as the insulin receptor pathway and the PI3K cascade, which revealed significantly altered protein phosphorylation during infection (Fig 6D). mTORC1 mediated signaling was also identified, which included members of the translation machinery. mRNA processing was also affected; in fact, mTORC1 was previously shown to modulate mRNA splicing through activated S6K interactions with the exon junction complex protein SKAR, which enhances spliced mRNA translation [67]. Notably, proteins aligning with the FAS signaling pathway had altered phosphorylation during influenza virus infection suggesting that mTORC1 has a role in regulating Fas-mediated apoptotic cell death during influenza virus infection. Indeed, Fas and FasL are upregulated during influenza virus infection [68–70], and the resulting apoptosis can promote viral replication [68,71]. Overall, our findings implicate mTORC1 as a central player in the regulation of influenza virus infection by altering the phosphorylation state of members of key signaling pathways.

## Discussion

**Fig 7:**
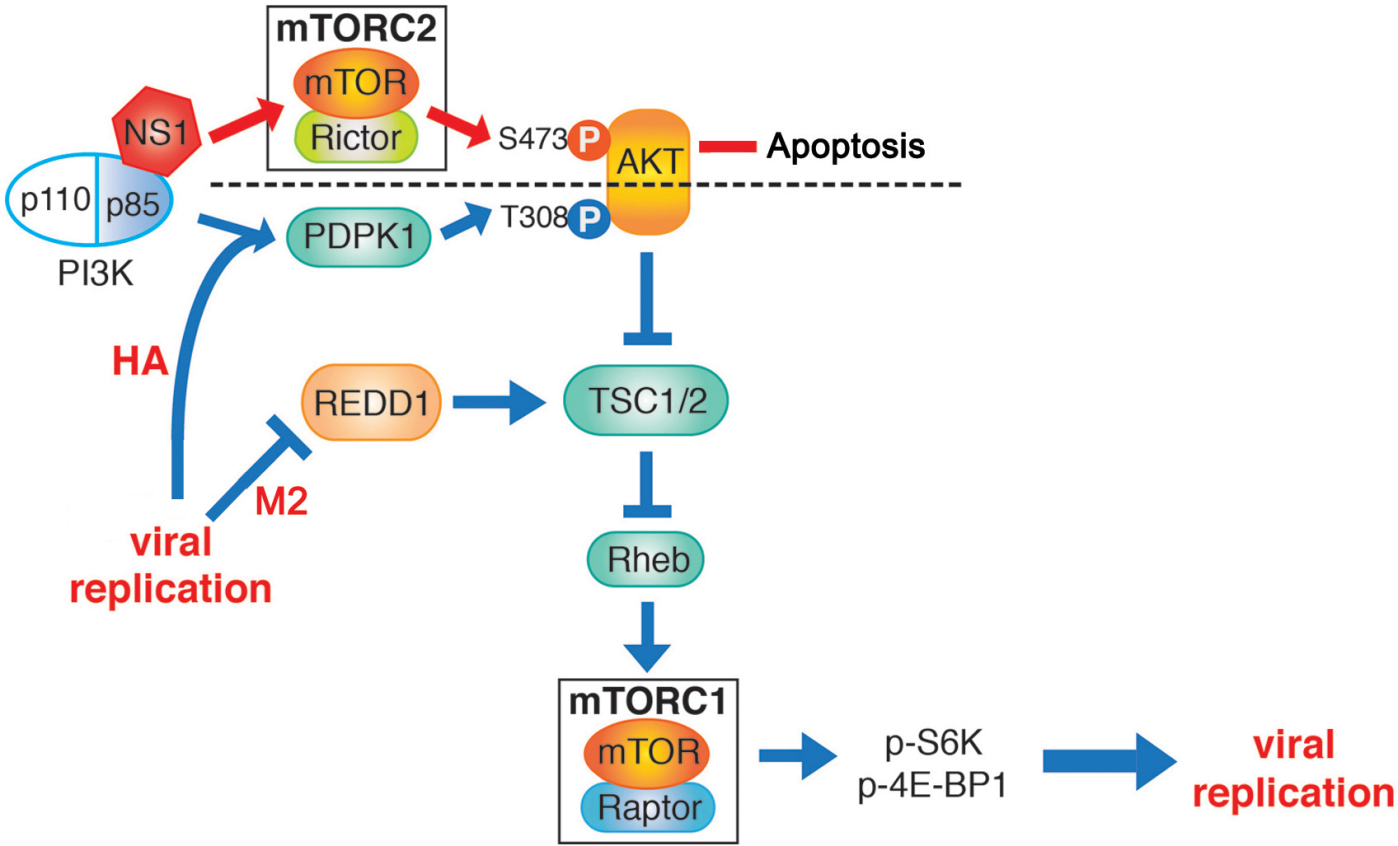
Schematic representation of the model for mTOR activation by influenza virus midway through the virus life cycle. The viral protein HA and virus replication promote mTORC1 activation through PDPK1-mediated phosphorylation of AKT at T308. In addition, down-regulation of REDD1 by the viral M2 protein amplifies or support mTORC1 activation downstream of AKT. NS1 promotes AKT phosphorylation at S473 via mTORC2 and this process is known to regulate apoptosis. Differential AKT phosphorylation dictates downstream effects.

The regulation of mTOR by influenza virus has not been previously characterized, although mTOR promotes influenza virus replication [4,5]. mTOR is central to protein production and cell survival, and therefore, it is understandable that influenza virus would exploit mTOR to regulate cellular processes for its advantage. The data presented herein have revealed several new findings regarding manipulation of mTOR signaling by influenza virus, which are summarized in Fig 7. We discovered that mTORC2 and PDPK1 are required during influenza virus infection to phosphorylate AKT, while AKT is required for influenza virus to induce mTORC1 signaling. PDPK1-mediated phosphorylation of AKT T308 is necessary for influenza virus to activate mTORC1, whereas mTORC2-mediated phosphorylation of AKT S473 is not required for mTORC1 activation. mTORC2 may contribute to the regulation of apoptosis by the viral NS1 protein during infection since previous reports have linked AKT S473 phosphorylation to NS1 and apoptosis [26]. Therefore, AKT is differentially phosphorylated by influenza virus to yield different outcomes related to viral protein expression and cell death.

We observed that mTORC1 activation by influenza virus required virus replication and promoted infection. The viral glycoprotein HA promotes mTORC1 activation while NS1 is dispensable. To this end, it has been shown that membrane accumulation of HA activates ERK signaling through protein kinase Cα to promote nuclear export of the viral genome [72]. In addition, HA expression can activate NF-κB via oxidative radicals and induction of IκB activity [73,74], which likely contributes to the regulation of immunity and inflammation during infection. Whether mTORC1 activation by HA contributes to these process remains to be determined and it would be an interesting topic for future investigation. Furthermore, we showed that influenza virus M2 protein down-regulates the mTORC1 inhibitor REDD1, a process that impacts the lates stages of infection. Since the viral M2 protein is a proton channel, M2-mediated mTORC1 activation might be analogous to the cellular v-ATPase proton channel that induces mTORC1 activation upon amino acid sensing and recruitment of scaffold proteins and regulators of mTORC1 [8].

REDD1 protein levels increase during viral entry and/or primary viral transcription and are then down-regulated at late stages of infection. The transient early increase in REDD1 levels likely represents an antiviral response, as REDD1 is a host defense factor [5]. REDD1 prevented induction of mTORC1 signaling by influenza virus. Inhibition of mTORC1 signaling by REDD1 during influenza virus infection was independent of AKT T308 dephosphorylation. Therefore, REDD1 acts downstream of AKT to exert its negative effects on mTORC1 signaling during influenza virus infection. It is likely that REDD1 targets the TSC1/2 complex possibly through promoting TSC2 activity, as previously described [49]. REDD1 has also been shown to induce dephosphorylation of AKT T308 to repress mTORC1 signaling in the absence of infection [50]. Perhaps REDD1 has the ability to down-regulate the mTOR pathway at several nodes, which would vary depending on the cellular environment or stimuli.

The impact of mTORC1 on influenza virus infected cells was also demonstrated with the identification of mTORC1-dependent substrates that were regulated during infection using a systematic phosphoproteomics approach. In addition to known mTORC1 substrates, we have also identified multiple mTORC1 substrates that were not previously revealed in an mTORC1 substrate screen using insulin stimulus [58], which could indicate that influenza virus modulates mTORC1 differently than insulin signaling. However, the differences could also be a result of the technical differences between the experiments. For example, the prior screen used *TSC2*^*+/+*^ and ^*-/-*^ MEFs in which they inhibited mTORC1 using rapamycin and Ku-0063794, they performed Stable Isotope Labeling with Amino acids in Cell culture (SILAC) to distinguish peptides from the differing conditions and their method of phosphopeptide enrichment was different than the one we used [58]. Rps6ka3, known as RSK2, is a known regulator of mTORC1 signaling [75], which is downstream in the MEK/ERK pathway that can stimulate mTORC1 [76]. Here, it appears that RSK2 is an mTORC1 substrate during influenza virus infection, which suggests that mTORC1 may regulate MEK/ERK signaling. This may be advantageous for influenza virus as MEK/ERK signaling is critical for viral replication [77]. We also observed that three members of the Fas signaling pathway had altered phosphorylation during influenza virus infection indicating that mTORC1 may influence Fas-mediated apoptosis during infection. FasL expression and apoptosis enhance influenza virus replication [68,71] and promote influenza virus pathogenicity in mice [78]. In addition, we identified key translation factors, eIF4B(S422) and eIF4G1(S1189), whose phosphorylation are regulated during infection in a mTORC1-dependent manner. Phosphorylation of eIF4B at S422 activates translation initiation upon amino acid refeeding via mTORC1 [79]. The mTORC1-mediated regulation of key constituents of the translation machinery during infection shown here is in agreement with its activity in promoting viral protein expression and viral replication. Taken together, these results indicate that mTORC1 signaling supports viral replication through regulation of translation and /or through Fas-mediated signaling and apoptotic responses to infection.

mTORC1 regulates protein translation and other cellular processes, such as autophagy and lipid biosynthesis [7]. Since these processes can influence influenza virus replication, trafficking of viral RNA/proteins and/or virion biogenesis, they may be useful for developing therapeutics and vaccines. Targeting host proteins required for viral replication is an alternative therapeutic strategy to avoid the rapid development of drug resistant viruses. Furthermore, promoting mTORC1 activation to enhance virus replication could have commercial implications for processes requiring robust virus replication, such as vaccine production. In sum, the activation of the mTORC1 pathway by influenza viruses midway through the virus replication cycle likely promotes mechanisms that antagonize cellular stress responses, such as translational shutoff and/or proper regulation of apoptosis to ensure an optimal environment for viral gene expression and progeny virion generation at later stages of the virus replication cycle.

## Materials and methods

### Cell culture

Human lung adenocarcinoma epithelial cells (A549, ATCC), Madin-Darby canine kidney cells (MDCK, ATCC), African green monkey kidney epithelial cells (Vero, ATCC), mouse embryonic fibroblasts (MEFs, already-existing collection from our laboratory [5]), human bone osteosarcoma cells with inducible expression of REDD1 (U2OS-REDD1, obtained from James Brugarolas, UT Southwestern) [80], human colon carcinoma cells (HCT116 *Pdpk*^*+/+*^ and ^*-/-*^ and *Akt*^*+/+*^ and ^*-/-*^, obtained from Bert Vogelstein, Johns Hopkins University) [81], and MDCK cells stably expressing influenza virus HA or M2 protein were cultured in DMEM containing 10% FBS (Atlas Biologicals or Sigma) and penicillin-streptomycin (Gibco). In the case of M2 expressing MDCK cells, 5 μM amantadine was added in the culture medium but removed 2 h prior to harvesting the cells. The human bronchiolar epithelial cells (HBEC30KT, a kind gift from Michael White, UT Southwestern) were grown in Keratinocyte-SFM supplemented with human recombinant epidermal growth factor (Invitrogen), bovine pituitary extract (Invitrogen) and penicillin-streptomycin. Primary wild-type MEFs were isolated from 129P2/OlaHsd mouse embryos (day 14) by mincing and trypsinization (after removal of the head), and cells were plated and used for experiments prior to senescence (approximately 8–10 passages). *Rictor*^*+/+*^ and ^*-/-*^ were kindly provided by Mark Magnuson [82]. HCT116 *Pdpk1*^*+/+*^ and ^*-/-*^ and *Akt*^*+/+*^ and ^*-/-*^ were graciously shared by Bert Vogelstein [81]. *Tbk1*^*+/+*^ and ^*-/-*^ MEFs were kindly provided by Tak Mak [83]. *Mavs*^*+/+*^ and ^*-/-*^ MEFs were generated as previously described [84]. Immortalized *Ifitm3*^*+/+*^ and ^*-/-*^ MEFs were generated from day 15 embryos according to published protocols [85]. *Atg5*^*+/+*^ and ^*-/-*^ MEFs were kindly provided by Beth Levine (UT Southwestern Medical Center, TX). Cells were maintained at 37°C with 5% CO_2_. Cells tested negative for mycoplasma. For tetracycline induction of REDD1 in U2OS-REDD1 cells, cells were pre-treated for 2 h with 1 μg/mL of tetracycline and throughout the infection.

### Reagents

Chemical reagents include: Torin1 (Tocris, in DMSO), HEPES (Gibco), urea (Sigma), PhoSTOP phosphatase inhibitor cocktail tablets (Roche), Complete EDTA-free protease inhibitor tablets (Roche), Trichloroacetic acid solution (Sigma), DMSO (Sigma), BX795 (gift from Michael White, UT Southwestern) and Tetracycline (USB Corporation). Antibodies used in western blots to detect viral proteins were generated against Influenza A virions (Meridian Life Science B65141G) (recognize HA, NP, M1, low level NA), HA (Genetex GTX127357), NP (Abcam ab20343), NA (GeneTex GTX125974), PA (GeneTex GTX 118991), PB1 (Santa Cruz sc-17601), PB2 (Santa Cruz sc-17603), M2 (clone 14C2, Thermo MA1-082), NS1 (generated by García-Sastre laboratory) and VSV M protein generated by our laboratory in collaboration with Cocalico Biologicals against full-length VSV M. Antibodies from Cell Signaling Technologies for western blot analysis were against phospho-S6K(T389) (#9234), S6K (#9202), phospho-4E-BP1(T37/46) (#2855), 4E-BP1 (#9644), phospho-Akt (S473, T308) (#4060, #9275), Akt1 (#2967), TBK1 (#3504), PDK1 (#5662) and p-eIF4B(S422) (#3591). Additional antibodies used for western blot analysis were against Rictor (Millipore 05–1471), IFITM3 (R&D Systems AF3377), MAVS (generated by Z. Chen laboratory), β-actin (Sigma A5441), REDD1 (Novus Biologicals NBP1-22966), ATG5 (Novus Biologicals NB110-53818), ATG7 (Sigma A2856), and LC3 (Novus Biologicals NB100-2220). Horseradish peroxidase (Hrp)-conjugated secondary antibodies used were donkey anti-rabbit and sheep anti-mouse (GE Healthcare NA934V and NA931V, respectively), as well as donkey anti-goat (Jackson Immunoresearch 705–035003). NA was expressed from a pCAGGS-NA plasmid.

### Viruses and infections

All virus work was performed in strict accordance with CDC guidelines for biosafety level 2 and 3 agents: BSL2 (A/WSN/1933, A/PR/8/1934 and rSh/1) and BSL3 (A/Shanghai/1/2013). All Sh/1 (A/Shanghai/1/2013, H7N9) virus work was performed in the BSL3 laboratory of the Icahn School of Medicine at Mount Sinai, NY.

Virus strains used in these studies were VSV-GFP and the following influenza viruses A/WSN/1933 (WSN), A/PR/8/1934 (PR8), A/Shanghai/1/2013 (Sh/1), recombinant A/Shanghai/1/2013 (rSh/1, "rSh/1 (6+2)": recombinant virus containing A/Shanghai/1/2013 segments 1, 2, 3, 5, 7 and 8 and A/PR/8/1934 segments 4 and 6) [39], PR8:WSN and PR8:WSNDeficientM2 [41]. Influenza viruses were propagated in chicken embryonated eggs or MDCK cells. A/WSN/1933 and A/PR/8/1934 viruses lacking NS1 were propagated as previously described [86,87]. To amplify stocks in MDCKs, cells were infected at an MOI of 0.01–0.001 in infection media: EMEM (ATCC, 30–2003), 10 mM HEPES (Gibco), 0.125% BSA (Gibco), 0.5 ug/mL TPCK trypsin (Worthington Biomedical Corporation). After 1 hour at 37°C, cells were washed and overlaid with infection media. Once cytopathic effect was evident around 48–72 hours post-infection, supernatants were harvested, centrifuged at 1,000 *x g* for 10 minutes, aliquoted and stored at -80°C.

For high MOI infection experiments (MOI of 2), adherent cells were serum starved overnight in infection media. EMEM infection media (see above) was used for A549, HBEC, U2OS-REDD1, Vero cells and primary, *Atg5*^*+/+/-/-*^ and *Ifitm3*^*+/+*^*/*^*-/-*^ MEFs. DMEM infection media (DMEM substituted for EMEM in standard infection media) was used for *Rictor*^*+/+*^/^*-/-*^, *Mavs*^*+/+*^/^*-/-*^ as well as *Tbk1*^*+/+*^*/*^*-/-*^ MEFs and HCT116 *Pdpk*^*+/+*^*/*^*-/-*^ and *Akt*^*+/+*^/^*-/-*^ to maintain cell viability. *Rictor*^*-/-*^ MEFs were plated at a density of 25% more than *Rictor*^*+/+*^ MEFs for each experiment to standardize the number of cells so that the same amount were being infected, as determined by cell counting prior to the infection. Nearly confluent cells in 12- or 24-well plates were mock infected with media only or infected with 150–200 μL virus in infection media for 1 hour at 37°C. After 1 hour, 350–800 μL of infection media was added and cells were incubated at 37°C prior to harvest. For Torin1 treated cells at a high MOI, cells were infected for 1 h at 37°C, after which the infection media was removed and media containing 250 nM Torin1 or 0.1% DMSO was added for the additional time indicated in the legend. Viral titer analyses by plaque assays were performed as described previously [88] and as indicated in the figure legends.

For western blot analysis, cells were washed with PBS and harvested in 2X sample buffer (125mM Tris HCl pH 6.8, 20% glycerol, 4% SDS), boiled 10 minutes and subjected to western blot analysis. Sample loading was standardized by Bradford assay (Biorad DC Protein Assay Kit). Western blot quantification was performed using ImageJ64. Images were converted to grayscale, rectangles were drawn around the lane of bands of interest and a profile plot was generated for each lane of bands. Each band was separated using the line tool, and the density of each band was determined by the area under the peak using the wand tool. Phosphorylated protein bands were compared to the total non-phosphorylated protein bands. Values were normalized to uninfected controls as indicated.

Cell viability assays (CellTiter-Glo, Promega) were performed to assess cell health. In Fig 5B, the MTT assay [Cell Proliferation Kit I (MTT), Roche) was used to determine cell survival, following the manufacturer’s protocol. For statistical analyses from three independent experiments, an unpaired, two-tailed *t*-test was performed. A normal distribution can be assumed for all populations (*p*>0.05).

To UV inactivate WSN, virus was exposed 10 cm from the UV lamp in the laminar flow hood for 7 minutes. The amount of virus inactivated was the same amount as non-inactivated virus used in each experiment for an MOI of 2 PFU/cell. Inactivation was confirmed by plaque assay, and a hemagglutination (HA) assay was performed following the VIRAPUR HA Assay Protocol to ensure UV-inactivated virus was present and still able to fuse with turkey red blood cells (LAMPIRE Biological Products).

### Mini-genome assay

U2OS or A549 cells were forward transfected in duplicates in 12 well plates with 450 ng pcDNA3-NP, 283.3 ng pcDNA3-PA, 283.3 ng pcDNA3-PB1 and 283.3 ng pcDNA3-PB2 +/- 200 ng spLuc using 3.2 μL/well Mirus TransIT-X2 transfection reagent (MIR6004) following manufacturer's protocol. For the experiment in Fig 3B, cells were serum starved overnight the following day. On day two post-transfection, the cells receiving FBS were stimulated with 10% FBS for 2 hours. The cells were harvested at 48 hours post-transfection in 2X sample buffer for western blot analysis or 1X Reporter Lysis Buffer (RLB, Promega, #E3971) to proceed with luciferase analysis. The Promega Luciferase Assay System (#E4030) was used to measure luciferase in each sample. Cells were suspended in 100 μL RLB, and 20 μL were mixed with 100 μL Luciferase Assay Reagent (Promega) following manufacturer's protocol. Luciferase was read on a BMG Labtech PHERAstar, and data were graphed after normalizing the reads to the pcDNA3-NP, -PA, -PB1 and -PB2 ΔspLuc wells to 1.

### RNA interference

For influenza virus mRNA-specific siRNAs, A549 cells were reverse transfected using RNAiMAX (Lipofectamine, Life Technologies) with 50 nM of a pool of 3 mRNA-specific siRNAs for approximately 36 h, during which cells were serum starved overnight prior to infection the next day as outlined above. Cell viability analysis (CellTiter-Glo, Promega) was performed to ensure that the siRNA concentrations were not toxic. Influenza virus mRNA-specific siRNAs were generated by Thermo Scientific to target mRNAs from the WSN strain (siGENOME modifications). siRNA sequences are as follows:

siRNA Universal Negative Control #1 and #2 (Sigma SIC001 and SIC002, proprietary sequences)

siGENOME Non-Targeting (pooled for use in experiments):

#2 5'-UAAGGCUAUGAAGAGAUACdTdT-3' (Dharmacon D-001210-02)
#3 5'-AUGUAUUGGCCUGUAUUAGdTdT-3' (Dharmacon D-001210-03)

Influenza virus (WSN)-specific (Table 1):

**Table 1:** 

siRNA	Gene ID	Sequence (5’-3’)	References (where applicable)
NP-1496	DQ508890	GGAUCUUAUUUCUUCGGAGdTdT	Ge Q et al., 2003 (DOI:10.1073); Sui HY et al., 2009 (DOI:10.1371)
NP-204	DQ508890	GCUUAACAAUAGAGAGAAUdTdT	
NP-694	DQ508890	CAACAUUCUCAAAGGGAAAdTdT	
PA-2087	CY034137	GCAAUUGAGGAGUGCCUGAdTdT	Ge Q et., 2003 (DOI:10.1073)
PA-381	CY034137	GGAGAGAAGUUCACAUAUAdTdT	
PA-1275	CY034137	GGAUAGAGCUCGAUGAGAUdTdT	
PB1-2257	CY034138	GAUCUGUUCCACCAUUGAAdTdT	Ge Q et., 2003 (DOI:10.1073)
PB1-143	CY034138	ACACAUCAGUACUCAGAAAdTdT	
PB1-1067	CY034138	GGUACAUGUUUGAGAGCAAdTdT	
PB2-2239	CY034139	ACGGAACUCUAGCAUACUUdTdT	
PB2-220	CY034139	GGACAAACUUUAUGGAGUAdTdT	
PB2-146	CY034139	GGAUGAUGGCAAUGAAAUAdTdT	
M1-598	L25828.1	UGGCUGGAUCGAGUGAGCAdTdT	Ge Q et., 2003 (DOI:10.1073)
M1-158	L25828.1	UGGCUAAAGACAAGACCAAdTdT	
M1-512	L25828.1	AGGCAAAUGGUGACAACAAdTdT	
M2-20	L25828.1	UCGAAACGCCUAUCAGAAAdTdT	
M2-219	L25828.1	GGAAGAAUAUCGAAAGGAAdTdT	
M2-237	L25828.1	ACAGCAGAAUGCUGUGGAUdTdT	Sui HY et al., 2009 (DOI:10.1371)
HA-563	DQ508905	GAAAGAAGUCCUUGUACUAdTdT	
HA-889	DQ508905	CGUCAAUGCAUGAGUGUAAdTdT	
HA-1065	DQ508905	GGGAUGGACUGGAAUGAUAdTdT	
NA-554	CY034134	GAGCAGUGGCUGUAUUAAAdTdT	
NA-1011	CY034134	UGGUAAUGGUGUUUGGAUAdTdT	
NA-1170	CY034134	CGUUCAACAUCCUGAGCUAdTdT	

### Quantitative RT-PCR

Cells were washed with PBS, harvested in TRIZOL (Invitrogen) and subjected to RNA extraction using Direct-zol RNA Miniprep Kit (Zymo Research). For each sample, 500 ng of RNA were used to generate cDNA by reverse transcription using the iScript cDNA Synthesis Kit (Bio-Rad) following manufacturer's protocol. cDNA was diluted 1:3, mixed with primers (300 nM total per reaction) and Roche 480 SYBR Green I Master real-time PCR reagents following manufacturer's protocol and subjected to quantitative real-time PCR using the Roche LightCycler 480. Real-time PCR was performed at 95°C for 5 min, 40 cycles of 95°C for 15 sec, 60°C for 15 sec and 72°C for 18 sec. A melting curve cycle was performed from 65°C to 95°C for quality assurance. Primers (Eurofins) used were REDD1 (forward: 5'- GACAGCAGCAACAGTGGCTTCG -3', reverse: 5'- GCTGCATCAGGTTGGCACAC -3'); 18S rRNA (forward: 5'- GTAACCCGTTGAACCCCATT -3', reverse: 5'- CCATCCAATCGGTAGTAGCG -3'); Minigenome RNA (firefly luciferase RNA) (forward: 5’-gaggttccatctgcaggta-3’, reverse: 5’-ccggtatccagatccacaac-3’). Primers to quantify viral mRNAs were previously described [88]. For statistical analyses, an unpaired, two-tailed *t*-test was performed. A normal distribution can be assumed for all populations (*p*>0.05).

### Phosphoproteomics screen

#### Infection and harvest

*Rictor*^*-/-*^ MEFs were plated in 15 cm plates (6 per condition), serum starved overnight in DMEM infection media and, at near confluency, were mock- or WSN-infected the following day. Cells were infected at an MOI of 2 PFU/cell for 1 h at 37°C. Infection media was removed after 1 h, and media containing 250 nM Torin1 or 0.1% DMSO was added for an additional 6 h. After 7 hpi, cells were washed twice with cold PBS and lysed in 1.5 mL/plate fresh urea lysis buffer (20 mM Hepes pH 8.0, 9M urea, 1X phosphatase inhibitor cocktail and 1X protease inhibitor cocktail). Cell lysates were sonicated 12 times for 10 second pulses with 30 second cooling periods between each pulse. Lysates were then placed on ice for 30 min with vortexing every 10 min. Cell debris was removed by centrifugation at 4°C for 10 minutes at 13,000 x *g*. Supernatants were collected and subjected to TCA precipitation. One volume of TCA solution (Sigma) was added to four volumes of cell extract and incubated on ice for 10 minutes. Proteins were collected by centrifugation at 14,000 rpm for 5 minutes at 4°C. Supernatants were removed, and the protein pellet was washed twice with 500 μL of cold acetone. Pellets were vacuum-dried using a Labconco CentriVap Concentrator for 20 minutes and stored at -20°C. Samples were prepped, labeled, phosphopeptide enriched and analyzed by LC-MS/MS by PTM Biolabs (Chicago, IL) using the methods below.

#### Sample preparation, labeling and phosphopeptide enrichment

Proteins were dissolved in buffer (8 M urea, 100 mM TEAB, pH 8.0) and the protein concentration was determined with 2-D Quant kit (GE Healthcare) according to the manufacturer’s instructions. For digestion, the protein solution was reduced with 10 mM DTT (Sigma) for 1 h at 37°C and alkylated with 20 mM IAA (iodoacetamide, Sigma) for 45 min at room temperature in darkness. For trypsin digestion, the protein sample was diluted by adding 100 mM TEAB to urea concentration less than 2 M. Finally, trypsin (Sequencing Grade Modified Trypsin, Promega) was added at 1:50 trypsin-to-protein mass ratio for the first digestion overnight and 1:100 trypsin-to-protein mass ratio for a second 4 h digestion.

After trypsin digestion, peptides were desalted by Strata X C18 SPE column (Phenomenex) and vacuum-dried. Peptides were reconstituted in 0.5 M TEAB and processed according to the manufacturer's protocol for 8-plex iTRAQ kit (AB Science). Briefly, one unit of iTRAQ reagent (defined as the amount of reagent required to label 100 μg of protein) were thawed and reconstituted in ACN (acetonitrile, Fisher Chemical). The peptide mixtures were then incubated for 2 h at room temperature, pooled, desalted and dried by vacuum centrifugation. The sample was then fractionated by high pH reverse-phase HPLC using Agilent 300Extend C18 column (5 μm particles, 4.6 mm ID, 250 mm length). Briefly, peptides were first separated with a gradient of 2% to 60% acetonitrile in 10 mM ammonium bicarbonate pH 10 over 80 minutes into 80 fractions. Then, the peptides were combined into 7 fractions and dried by vacuum centrifugation.

Peptide mixtures were first incubated with IMAC microspheres suspension with vibration. The IMAC microspheres with enriched phosphopeptides were collected by centrifugation, and the supernatant was removed. To remove nonspecifically adsorbed peptides, the IMAC microspheres were washed with 50% ACN/6% TFA (trifluoroacetic acid, Sigma) and 30% ACN/0.1% TFA, sequentially. To elute the enriched phosphopeptides from the IMAC microspheres, elution buffer containing 10% NH_4_OH was added and the enriched phosphopeptides were eluted with vibration. The supernatant containing phosphopeptides was collected and lyophilized for LC-MS/MS analysis.

### LC-MS/MS analysis

Peptides were dissolved in solvent A (0.1% FA (formic acid, Fluka) in 2% ACN) and directly loaded onto a reversed-phase pre-column (Acclaim PepMap 100, Thermo Scientific). Peptide separation was performed using a reversed-phase analytical column (Acclaim PepMap RSLC, Thermo Scientific) with a linear gradient of 4–22% solvent B (0.1% FA in 98% ACN) for 50 min, 22–35% solvent B for 12 min, 35–85% solvent B for 4 min and holding at 85% for the last 4 min at a constant flow rate of 300 nl/min on an EASY-nLC 1000 UPLC system. The resulting peptides were analyzed by Q Exactive^TM^ Plus Hybrid Quadrupole-Orbitrap Mass Spectrometer (ThermoFisher Scientific). The peptides were subjected to NSI source followed by tandem mass spectrometry (MS/MS) in Q Exactive^TM^ Plus coupled online to the UPLC. Intact peptides were detected in the Orbitrap at a resolution of 70,000. Peptides were selected for MS/MS using NCE setting as 28; ion fragments were detected in the Orbitrap at a resolution of 17,500. A data-dependent procedure that alternated between one MS scan followed by 20 MS/MS scans was applied for the top 20 precursor ions above a threshold ion count of 5.0E3 in the MS survey scan with 15.0s dynamic exclusion. The electrospray voltage applied was 2.0 kV. Automatic gain control (AGC) was used to prevent overfilling of the ion trap; 5E4 ions were accumulated for generation of MS/MS spectra. For MS scans, the m/z scan range was 350 to 1800. Fixed first mass was set as 100 m/z.

The resulting MS/MS data was processed using MaxQuant with integrated Andromeda search engine (v.1.4.1.2). Tandem mass spectra were searched against *SwissProt_Mouse* database concatenated with reverse decoy database. Trypsin/P was specified as cleavage enzyme allowing up to 2 missing cleavages, 5 modifications per peptide and 5 charges. Mass error was set to 10 ppm for precursor ions and 0.02 Da for fragment ions. Carbamidomethylation on Cys was specified as fixed modification and oxidation on Met, phosphorylation on Ser, Thr, Tyr and acetylation on protein N-terminal were specified as variable modifications. False discovery rate (FDR) thresholds for protein, peptide and modification site were specified at 1%. Minimum peptide length was set at 7. For quantification method, iTRAQ-8 plex was selected. All the other parameters in MaxQuant were set to default values. The site localization probability was set as > 0.5. The resulting raw data can be found in S2 Table.

### Gene set enrichment analysis (GSEA) of phosphoproteomics data and selection of interferon regulated proteins

Protein phosphorylation site ratios were generated from the LC-MS/MS values by comparing WSN-infected + DMSO(control)-treated *Rictor*^*-/-*^ MEFs to WSN-infected + Torin1-treated *Rictor*^*-/-*^ MEFs (mTORC1 substrates). The fold-change for each phosphorylation site between groups was calculated. Protein sites that showed +/- 1.5-fold change or more in phosphorylation are shown (Fig 6A and S1 Table). The dataset was then subjected to Gene Set Enrichment Analysis (GSEA) (http://software.broadinstitute.org/gsea/index.jsp) to reveal the top pathways that showed the best enrichment scores. Interferon regulated genes were selected based on the database http://www.interferome.org [89].

### Ethics statement

The study using embryonated chicken was carried out in strict accordance with recommendations in the Guide for the Care and Use of Laboratory Animals of the National Institutes of Health. The use of embryonated chicken eggs before hatching is not considered animal use. Embryonated eggs were purchased from Charles River Laboratories, inoculated with influenza viruses at day 10, incubated at 37°C for 2 days, and then incubated at 4°C overnight before allantoid fluid harvesting.

## Supporting information

S1 Fig Western blot quantifications for Fig 1 and mTORC1 activation in Rictor knockdown cells. (**A-F**) Protein bands from the indicated blots from Fig 1 were quantified by ImageJ64 analysis. (**G**) A549 cells were transfected for 48 h with control siRNAs or siRNAs to knock down Rictor. Cells were then infected with WSN at MOI of 2 PFU/cell for 6h, 8h and 10h. Cell lysates were subjected to immunoblot analysis to detect the depicted proteins. (**H-M**) Protein bands from the indicated blots from Fig 1 were quantified by ImageJ64 analysis.
(TIF)

S2 Fig Western blot quantifications for Fig 2A. Protein bands were quantified by ImageJ64 analysis comparing (**A**) p-AKT(T308) to total AKT levels and (**B**) p-AKT(S473) to total AKT levels after normalizing to the mock-infected controls.
(TIF)

S3 Fig Diverse influenza virus strains activate mTORC1. (**A**) A549 cells were infected at MOI of 2 PFU/cell with WSN for the indicated times. (**B**) Primary MEFs or (**C**) HBEC30KT cells were infected with WSN for 6 h or 8 h, respectively, at MOI of 2 PFU/cell. A549 cells were infected with (**D**) Sh/1 (H7N9), (**E**) rSh/1 (recombinant Sh/1) and WSN (H1N1), (**F**) VSV-GFP, WSN (or treated with 5% serum for 7 h) for 6h at MOI of 2 PFU/cell. Immunoblot analyses were performed for detection of viral proteins (influenza virus M1 or VSV M) or host proteins (total and phosphorylated S6K and 4E-BP1). Total S6K serves as the loading control. The upper band in the S6K/p-S6K blots is p85 S6K, whereas the lower band is p70 S6K. Data are representative of three independent experiments.
(TIF)

S4 Fig Autophagy, M2, and IFN expression are not required for mTORC1 activation by influenza virus. (**A**) A549 cells were infected with wild-type PR8:WSN or PR8:WSNDeficientM2 at MOI of 2 PFU/cell for 6 h. (**B**) A549 cells were transfected with the indicated siRNAs for 48 h followed by infection with WSN at MOI of 2 PFU/cell for 6 h. (**C**) *Atg5*^*+/+*^ and *Atg5*^*-/-*^ MEFs were infected with WSN at MOI of 2 PFU/cell for 6 h. Immunoblot analyses were performed with antibodies against the depicted proteins. Total S6K serves as the loading control. Data are representative of three (**A**) or two (**B**,**C**) independent experiments. (**D**) UV inactivation of WSN. WSN was UV-inactivated for 7 minutes under UV light. WSN and UV-inactivated WSN (UV WSN) were subjected to both plaque assay and HA assay to confirm UV inactivation prior to infection by assessing infectious virus (PFU/mL) and quantifying virions (HA unit/50 μl). These assays were carried out each time WSN was UV-inactivated prior to infection. (**E**) Poly(I:C) stimulation does not induce mTORC1 activiation. MEFs were non-treated or treated with rapamycin (250nM) or Torin (250nM) and transfected with high molecular weight (HMW) poly(I:C) at 1 μg/ml for the indicated time points. Cell lysates were subjected to immunoblot analysis with the indicated antibodies. Mito70 was used as loading control. (**F**) As control for **E,** MEFs were also mock infected or infected with influenza A virus at MOI of 2 PFU/cell. Cell extracts were obtained at 8h post-infection and subjected to immunoblot analysis with the depicted antibodies. (**G**) MEFs were mock transfected or transfected with HMW poly(I:C) at 0.5 μg/ml for 6 and 12h. Total RNA was extracted at the indicated time points post-transfection and the relative abundance of mouse IFN β was measured by real time PCR. Data from triplicate experiments were normalized to β-Actin.
(TIF)

S5 Fig Quantification of Fig 4A. Western blots shown in Fig 4A were quantified and normalized to respective controls, as depicted in this figure, using the ImageJ64 analysis.
(TIF)

S6 Fig Cell viability at multiple times during Torin1 treatment and viral replication. (**A**) A549 cells were treated with 0.1% DMSO or 250 nM Torin1 for the indicated times. Cell viability was determined by measuring ATP levels and calculated as a percent of the DMSO control. (**B**) A549 cells were infected with WSN at MOI of 2 PFU/cell for 1 h and then treated with 250 nM Torin1 or DMSO for an additional 9 h. QPCR was performed to measure viral mRNA levels. Mean and SD are shown, *n* = 3, ***p<0.001. (**C**) A549 cells were infected for 24h with rSh/1 at MOI of 0.001 in the absence or presence of Torin. Viral titers were measured by plaque assay. Error bars are SEM, *n* = 9, **p<0.01. (**D**) A549 cells were transfected for 48 h with control siRNAs or siRNAs to knock down Rictor as in S1G Fig. Cells were then infected with WSN at MOI of 0.01 for 24h and 48h. Viral titers were measured by plaque assay. Error bars represent SD, *n* = 3, *p<0.05.
(TIF)

S1 Table mTORC1-dependent phosphorylation on proteins identified in mock-infected *Rictor*^*-/-*^ MEFs in the absence or presence of Torin1. The table indicates protein names and IDs, position(s) of amino acid (aa) phosphorylation, and the ratio of phosphopeptide reads (> and < 1.5 fold or more) in Torin1-treated vs. DMSO-treated cells.
(XLSX)

S2 Table Raw data from the proteomics studies described in the LC-MS/MS analysis section in Methods. (XLSX)

S1 Methods RNA interference, reagent, and poly(I:C) transfections described in supplementary figures. (DOCX)
