# Supplementary figures and images for: Influenza virus differentially activates mTORC1 and mTORC2 signaling to maximize late stage replication
Source: PLoS Pathog. 2017 Sep 27;13(9):e1006635. doi: 10.1371/journal.ppat.1006635 (PMC5617226; doi:10.1371/journal.ppat.1006635)

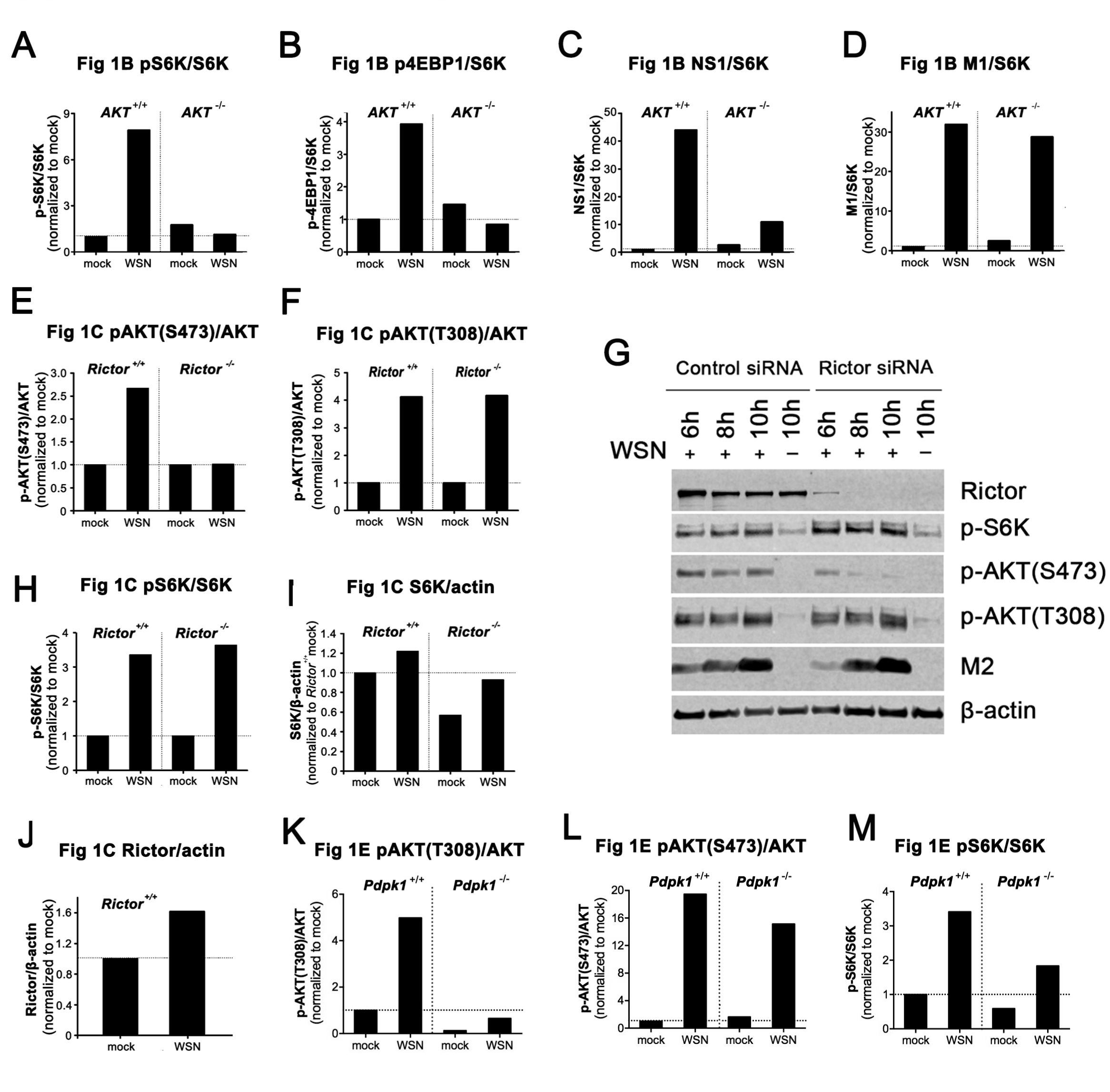

Supplement: S1 Fig — (A-F) Protein bands from the indicated blots from Fig 1 were quantified by ImageJ64 analysis. (G) A549 cells were transfected for 48 h with control siRNAs or siRNAs to knock down Rictor. Cells were then infected with WSN at MOI of 2 PFU/cell for 6h, 8h and 10h. Cell lysates were subjected to immunoblot analysis to detect the depicted proteins. (H-M) Protein bands from the indicated blots from Fig 1 were quantified by ImageJ64 analysis. (TIF) [file ppat.1006635.s001.tif]

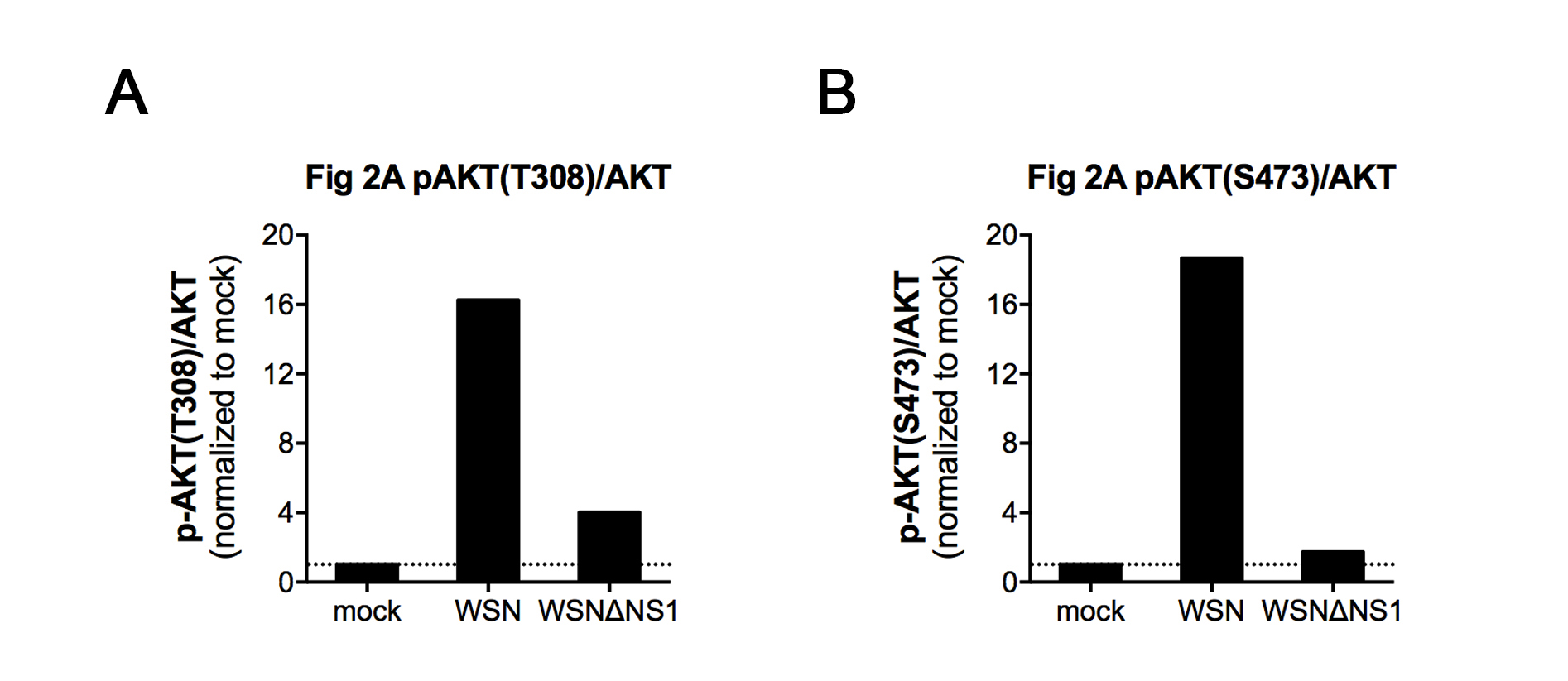

Supplement: S2 Fig — Protein bands were quantified by ImageJ64 analysis comparing (A) p-AKT(T308) to total AKT levels and (B) p-AKT(S473) to total AKT levels after normalizing to the mock-infected controls. (TIF) [file ppat.1006635.s002.tif]

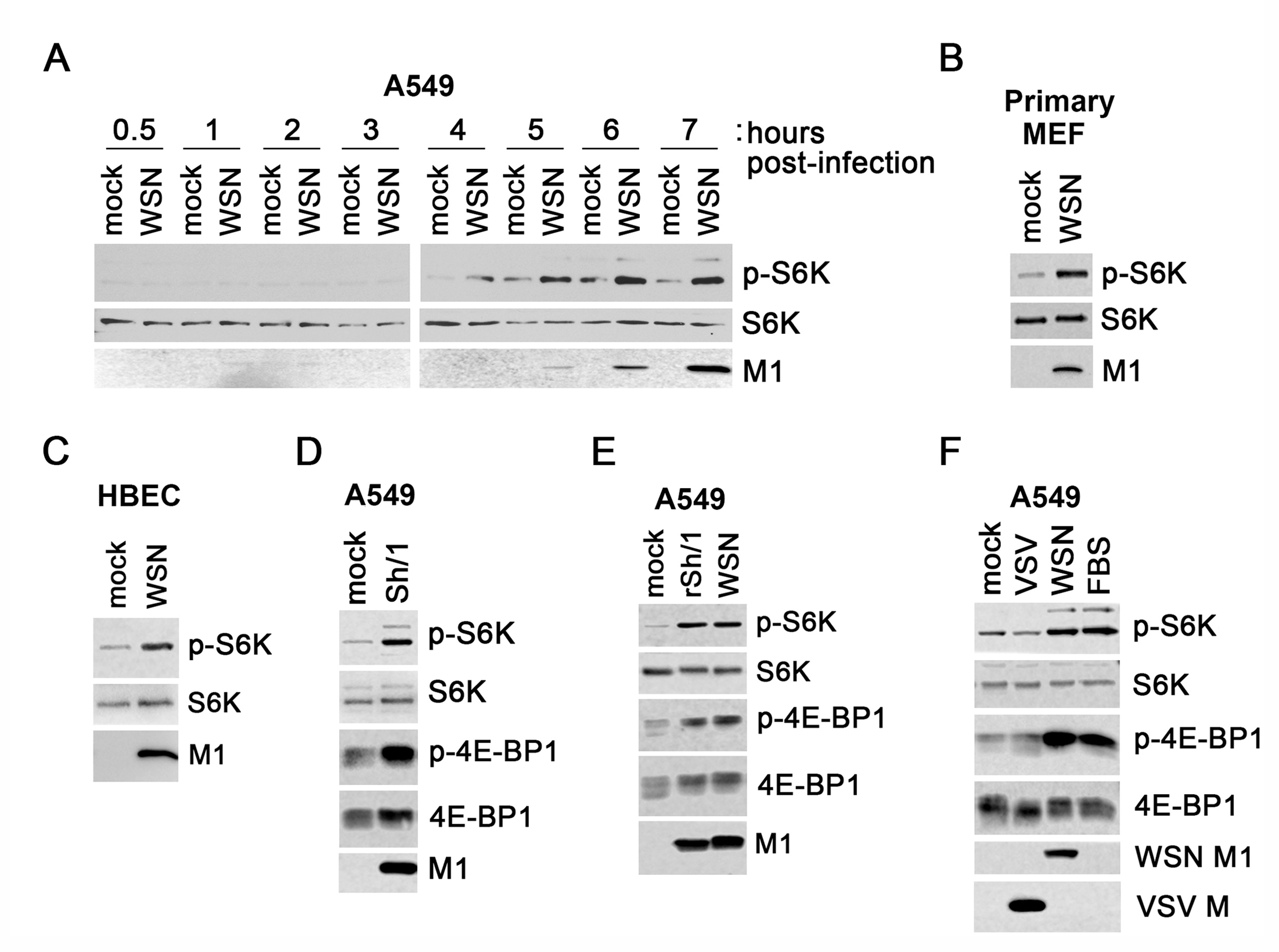

Supplement: S3 Fig — (A) A549 cells were infected at MOI of 2 PFU/cell with WSN for the indicated times. (B) Primary MEFs or (C) HBEC30KT cells were infected with WSN for 6 h or 8 h, respectively, at MOI of 2 PFU/cell. A549 cells were infected with (D) Sh/1 (H7N9), (E) rSh/1 (recombinant Sh/1) and WSN (H1N1), (F) VSV-GFP, WSN (or treated with 5% serum for 7 h) for 6h at MOI of 2 PFU/cell. Immunoblot analyses were performed for detection of viral proteins (influenza virus M1 or VSV M) or host proteins (total and phosphorylated S6K and 4E-BP1). Total S6K serves as the loading control. The upper band in the S6K/p-S6K blots is p85 S6K, whereas the lower band is p70 S6K. Data are representative of three independent experiments. (TIF) [file ppat.1006635.s003.tif]

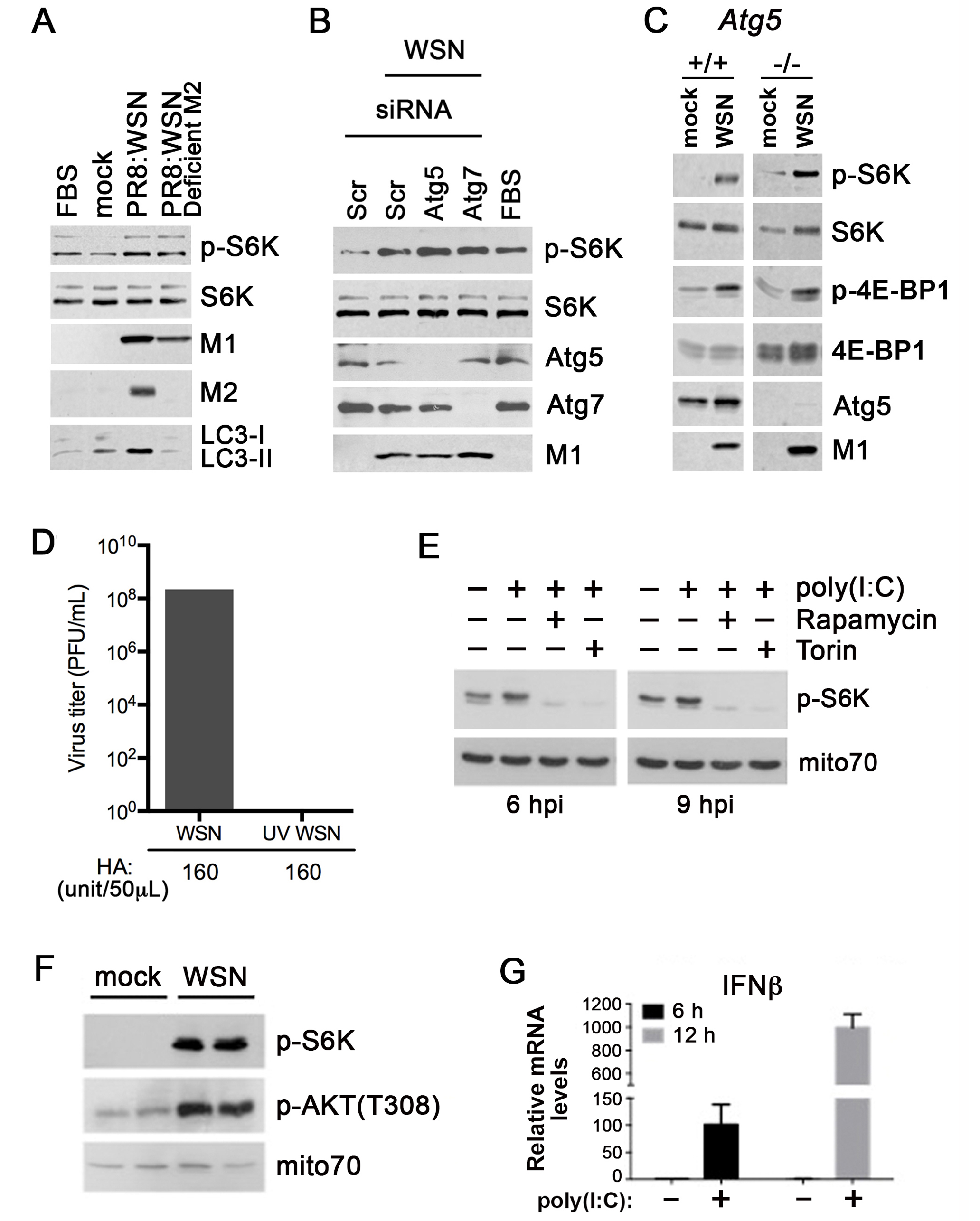

Supplement: S4 Fig — (A) A549 cells were infected with wild-type PR8:WSN or PR8:WSNDeficientM2 at MOI of 2 PFU/cell for 6 h. (B) A549 cells were transfected with the indicated siRNAs for 48 h followed by infection with WSN at MOI of 2 PFU/cell for 6 h. (C) Atg5+/+ and Atg5-/- MEFs were infected with WSN at MOI of 2 PFU/cell for 6 h. Immunoblot analyses were performed with antibodies against the depicted proteins. Total S6K serves as the loading control. Data are representative of three (A) or two (B,C) independent experiments. (D) UV inactivation of WSN. WSN was UV-inactivated for 7 minutes under UV light. WSN and UV-inactivated WSN (UV WSN) were subjected to both plaque assay and HA assay to confirm UV inactivation prior to infection by assessing infectious virus (PFU/mL) and quantifying virions (HA unit/50 μl). These assays were carried out each time WSN was UV-inactivated prior to infection. (E) Poly(I:C) stimulation does not induce mTORC1 activiation. MEFs were non-treated or treated with rapamycin (250nM) or Torin (250nM) and transfected with high molecular weight (HMW) poly(I:C) at 1 μg/ml for the indicated time points. Cell lysates were subjected to immunoblot analysis with the indicated antibodies. Mito70 was used as loading control. (F) As control for E, MEFs were also mock infected or infected with influenza A virus at MOI of 2 PFU/cell. Cell extracts were obtained at 8h post-infection and subjected to immunoblot analysis with the depicted antibodies. (G) MEFs were mock transfected or transfected with HMW poly(I:C) at 0.5 μg/ml for 6 and 12h. Total RNA was extracted at the indicated time points post-transfection and the relative abundance of mouse IFN β was measured by real time PCR. Data from triplicate experiments were normalized to β-Actin. (TIF) [file ppat.1006635.s004.tif]

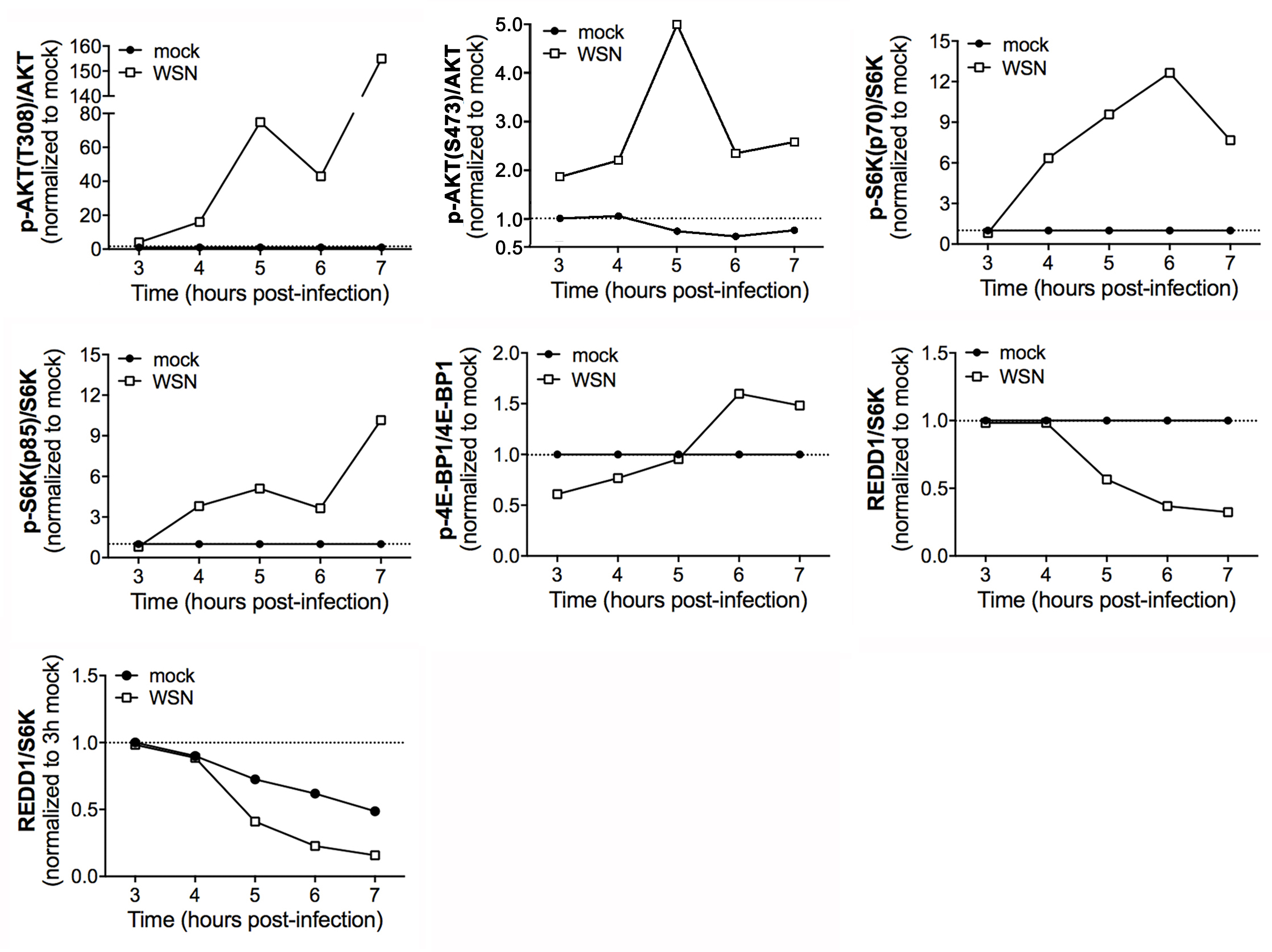

Supplement: S5 Fig — Western blots shown in Fig 4A were quantified and normalized to respective controls, as depicted in this figure, using the ImageJ64 analysis. (TIF) [file ppat.1006635.s005.tif]

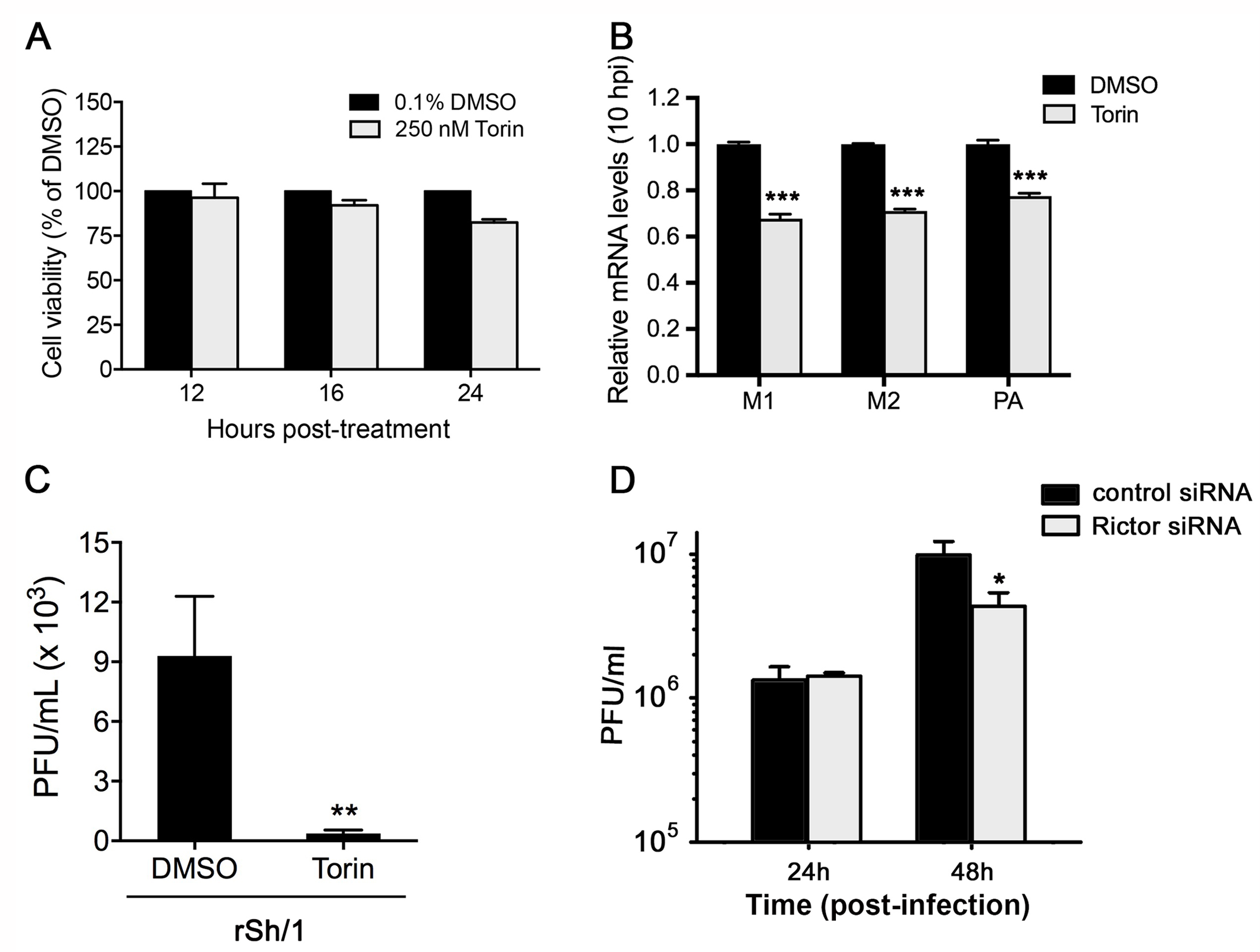

Supplement: S6 Fig — (A) A549 cells were treated with 0.1% DMSO or 250 nM Torin1 for the indicated times. Cell viability was determined by measuring ATP levels and calculated as a percent of the DMSO control. (B) A549 cells were infected with WSN at MOI of 2 PFU/cell for 1 h and then treated with 250 nM Torin1 or DMSO for an additional 9 h. QPCR was performed to measure viral mRNA levels. Mean and SD are shown, n = 3, ***p<0.001. (C) A549 cells were infected for 24h with rSh/1 at MOI of 0.001 in the absence or presence of Torin. Viral titers were measured by plaque assay. Error bars are SEM, n = 9, **p<0.01. (D) A549 cells were transfected for 48 h with control siRNAs or siRNAs to knock down Rictor as in S1G Fig. Cells were then infected with WSN at MOI of 0.01 for 24h and 48h. Viral titers were measured by plaque assay. Error bars represent SD, n = 3, *p<0.05. (TIF) [file ppat.1006635.s006.tif]
