# Supplementary material for: Influenza virus differentially activates mTORC1 and mTORC2 signaling to maximize late stage replication
Source: PLoS Pathog. 2017 Sep 27;13(9):e1006635. doi: 10.1371/journal.ppat.1006635 (PMC5617226; doi:10.1371/journal.ppat.1006635)
Supplement: S1 Methods — (DOCX) [file ppat.1006635.s009.docx]

**Supplementary Methods**

**RNA interference**

A549 cells were reverse transfected using RNAiMAX (Lipofectamine, Life Technologies) with 50 nM Rictor or control siRNAs for 48h prior to infection. Cells were mock or virus infected for 6h, 8h, 10h and then harvested in sample buffer for western blot analysis. Cell viability analysis (CellTiter-Glo, Promega) was performed to ensure that the siRNA concentrations were not toxic.

Rictor siRNAs (Sigma SASI_Hs02 00366683; SASI_Hs01 00223573)

**Reagent**

Monoclonal antibody against Complex II subunit 70 kD (Mito-70 kD) was obtained from Abcam (#ab14715).

**Poly(I:C) transfections**

Cells (3x10^5^) were plated in 6-well dish overnight in 2ml complete media. Next day, Lipofectamine 2000 – poly (I:C) complex was prepared: 50μl of serum/antibiotic free media was mixed with 4μl of Lipofectamine 2000. Serum/antibiotic free media (50μl) of was mixed with 2 μl of HMW poly(I:C) [1mg/ml] for immunoblot analysis or 1μl of HMW poly IC [1mg/mL] for QPCR analysis. Lipofectamine and poly (I:C)/RNA solutions were mixed for 20min at room temperature. The mixture was then added to the cells. After incubation, cells were washed twice with PBS and 2X lysis buffer was added for immunoblots or 1ml of TriZol for RNA isolation.
